# Establishing Covalent Organic Framework “A&B” Gel via Hydrogen Bond Exchange‐Induced Microphase Separation

**DOI:** 10.1002/advs.202508484

**Published:** 2025-08-19

**Authors:** Zhiwen Fan, Zihao Liao, Feng Zhang, Yang Huang, Chunyue Pan, Shuai Gu, Juntao Tang, Baosheng Wei, Jiayin Yuan, Guipeng Yu

**Affiliations:** ^1^ College of Chemistry and Chemical Engineering Central South University Changsha Hunan 410083 P. R. China; ^2^ Shenzhen Cham New Energy Battery Technology Co. Dongguan 523000 China; ^3^ Department of Materials and Environmental Chemistry Stockholm University Stockholm SE‐10691 Sweden

**Keywords:** covalent organic framework, gel electrolytes, self‐assembly

## Abstract

Rapid and controllable gelation of covalent organic framework (COF) materials with ordered accessible nanochannels is conducive to their use in versatile applications. However, challenges arise from intricate high‐rate polymerization processes and unregulated phase separation, which complicate the fine management on equilibrium morphology and crystallinity in COFs. Herein, inspired by prevalent hydrogen bonding interactions in nature, a hydrogen bond exchange (HBE)‐induced microphase separation (termed HBEiMS) pathway is proposed for the facile preparation of hydrazone‐linked COF “A&B” gels. The split‐prepared Glue A and Glue B avoid aggregation formation and precipitation of the powdered products after non‐homogeneous phase nucleation following the rapid reaction of monomers in a one‐pot manner. The phase transformation induced by HBE enables homogeneous separation of bulk materials, resulting in successful preparation of highly crystalline COF gels in seconds at room temperature. Furthermore, the charge transport of oxygen atoms within the robust COF pores is better controlled by modifying the distal group of the monomer side chains, resulting in a high cycling stability of the gel electrolyte beyond 1600 h. Collectively, findings in this study can fuel future development of COF materials and their possible commercial use.

## Graphical abstract

Inspired by prevalent hydrogen bonding interactions in nature, a hydrogen bond exchange (HBE)‐induced microphase separation (termed HBEiMS) pathway is proposed for the facile preparation of hydrazone‐linked covalent organic framework (COF) “A&B” gels. The charge transport of oxygen atoms within the robust COF pores is better controlled by modifying the distal group of the monomer side chains, resulting in a high cycling stability of the gel electrolyte beyond 1600 h.

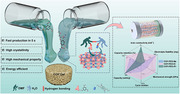

## 1 Introduction

Functional organic polymers, such as covalent organic frameworks, integrate the structural versatility of conventional polymers with the highly ordered crystalline architectures of advanced materials.^[^ ^1^, ^2^, ^3^, ^4^, ^5^, ^6^ ^]^ COFs with optimized electronic conductivity and abundant redox‐active sites exhibit enhanced electrochemical performance due to efficient charge transport and favorable redox kinetics.^[^ ^7^, ^8^, ^9^, ^10^ ^]^ The highly ordered nanoporous structure of COF promotes superior charge transport kinetics due to their well‐aligned pore channels, which provide optimized pathways for rapid ion migration and electron transfer.^[^ ^11^, ^12^, ^13^, ^14^ ^]^ As a result, COF‐based materials have emerged as promising candidates for next‐generation energy storage systems, including supercapacitors, lithium‐ion batteries, and related electrochemical devices.^[^ ^15^, ^16^, ^17^, ^18^ ^]^

Solution processability, which would improve the capacity for low‐cost processing without compromising on materials performance, is highly desired for the applications of the crystalline materials.^[^ ^19^, ^20^ ^]^ Bringing insoluble porous COF materials into scalable products for practical applications is crucial, while it remains a technological limit.^[^ ^21^ ^]^ This is mainly due to the fact that most COFs are synthesized via solvothermal or melt reactions.^[^ ^22^ ^]^ These syntheses are usually carried out in closed environments to maintain solvent autogenous pressure and heat to drive the rearrangement of monomers to achieve high crystallinity of the material, making it difficult to transform small‐scale experimental models into industrial production.^[^ ^23^ ^]^ In addition, the final network crystalline products are produced commonly as powders with low mechanical stability, which are clearly not ideal to meet end users.^[^ ^24^ ^]^

To enable molding and processing of covalent organic skeletons, strategies such as organic flux‐mediated synthesis and end‐group‐protected synthesis have been successively developed.^[^ ^25^, ^26^, ^27^ ^]^ The preparation of COFs has been accomplished by adding suitable fluxes or implementing group protection to slow down COF formation and modulate the nucleation rate.^[^ ^28^ ^]^ Although these methods enabled the preparation of COF gels, the cumbersome synthetic process and long reaction periods (>72 h) posed a serious intermediate obstacle in exploring different application scenarios of COFs.^[^ ^27^, ^29^ ^]^ Efficient and controllable synthesis of COF hinges on regulating the “dynamic covalent self‐assembly” of monomers, while balancing rapid polymerization and preventing unregulated phase separation of building blocks.^[^ ^30^ ^]^ Addressing this trade‐off represents a significant challenge, as achieving both high crystallinity and processability in COF materials has proven difficult. Existing studies have primarily focused on either: 1) developing rapid synthesis methods for highly crystalline COF powders,^[^ ^31^ ^]^ or 2) optimizing the gelation process for COF material molding, with limited success in simultaneously accomplishing both objectives.^[^ ^17^ ^]^ The direct preparation of COF‐functionalized gels remains a key bottleneck hindering their practical application.

**Scheme 1:**
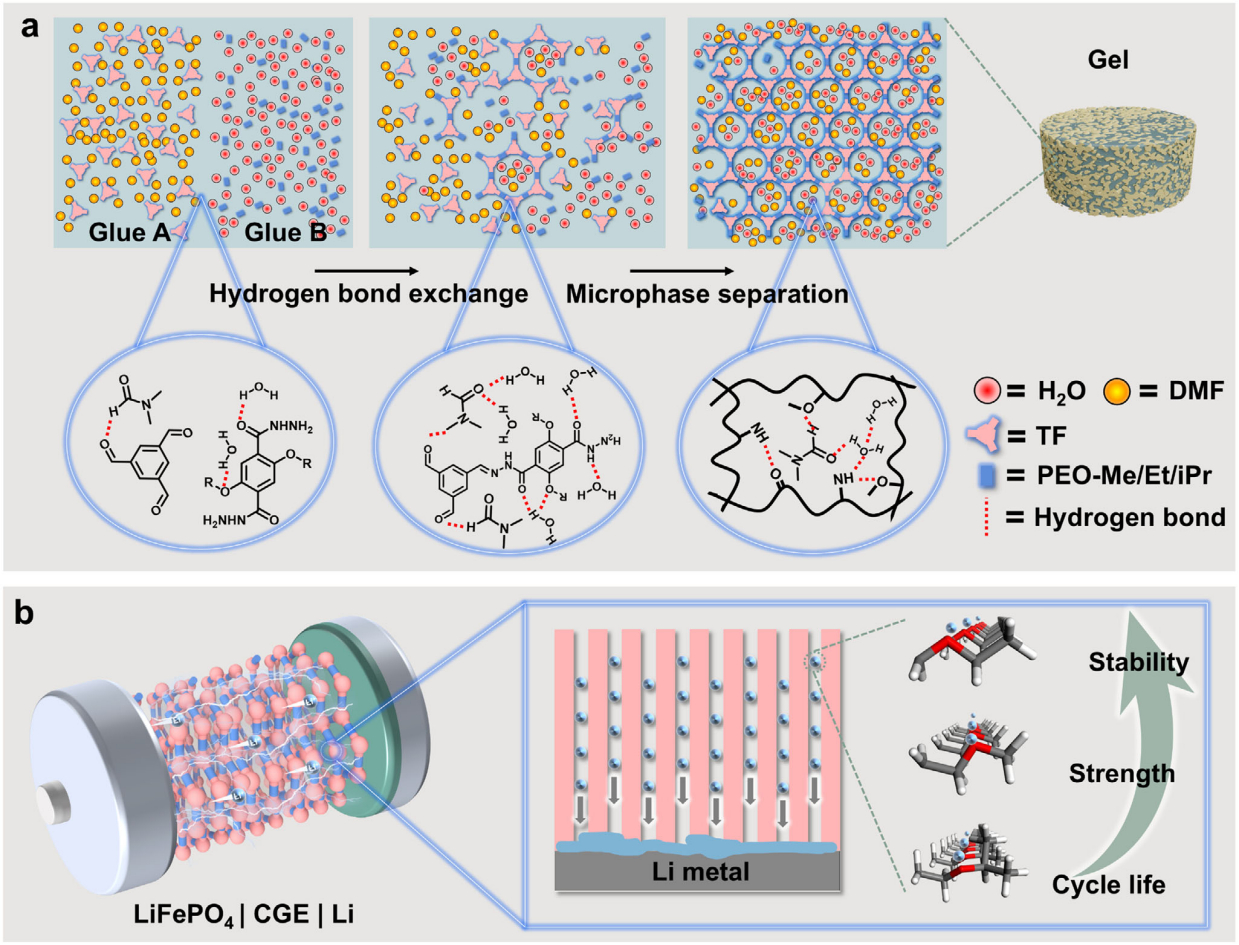
a) Hydrogen bond exchange‐induced microphase separation to construct covalent organic framework “A&B” gels. b) Side‐chain end‐groups modulate battery performance.

Herein, inspired by prevalent hydrogen bonding interactions in nature, we establish a hydrogen bond exchange (HBE)‐induced microphase separation strategy (termed HBEiMS) to prepare perylene‐bonded COF “A&B” gels facilely (**Scheme** **1**). The separately prepared Glue A and Glue B successfully avoided formation of aggregation and thus their precipitation as powder products generated otherwise in the one‐pot reaction manner. The dipole‐induced antiparallel stacking and the combination of intra‐ and interlayer hydrogen bonding by mixing A & B gels promoted crystallization of the hydrazone‐linked COF. HBEiMS stabilized the homogeneous phase separation of the COF bulk material and formed highly crystalline COF gels in seconds at room temperature. The developed gel electrolyte demonstrates exceptional cycling stability, maintaining stable performance over 1600 hours of continuous operation. Our rapid and heating‐free process reveals the key influence of hydrogen bonding of solvents in COF molding, and inspires scale‐up production and uses in turn.

## 2 Results and Discussion

### 2.1 Modulation of COF Expression by Hydrogen Bonding Interactions

**Figure 1:**
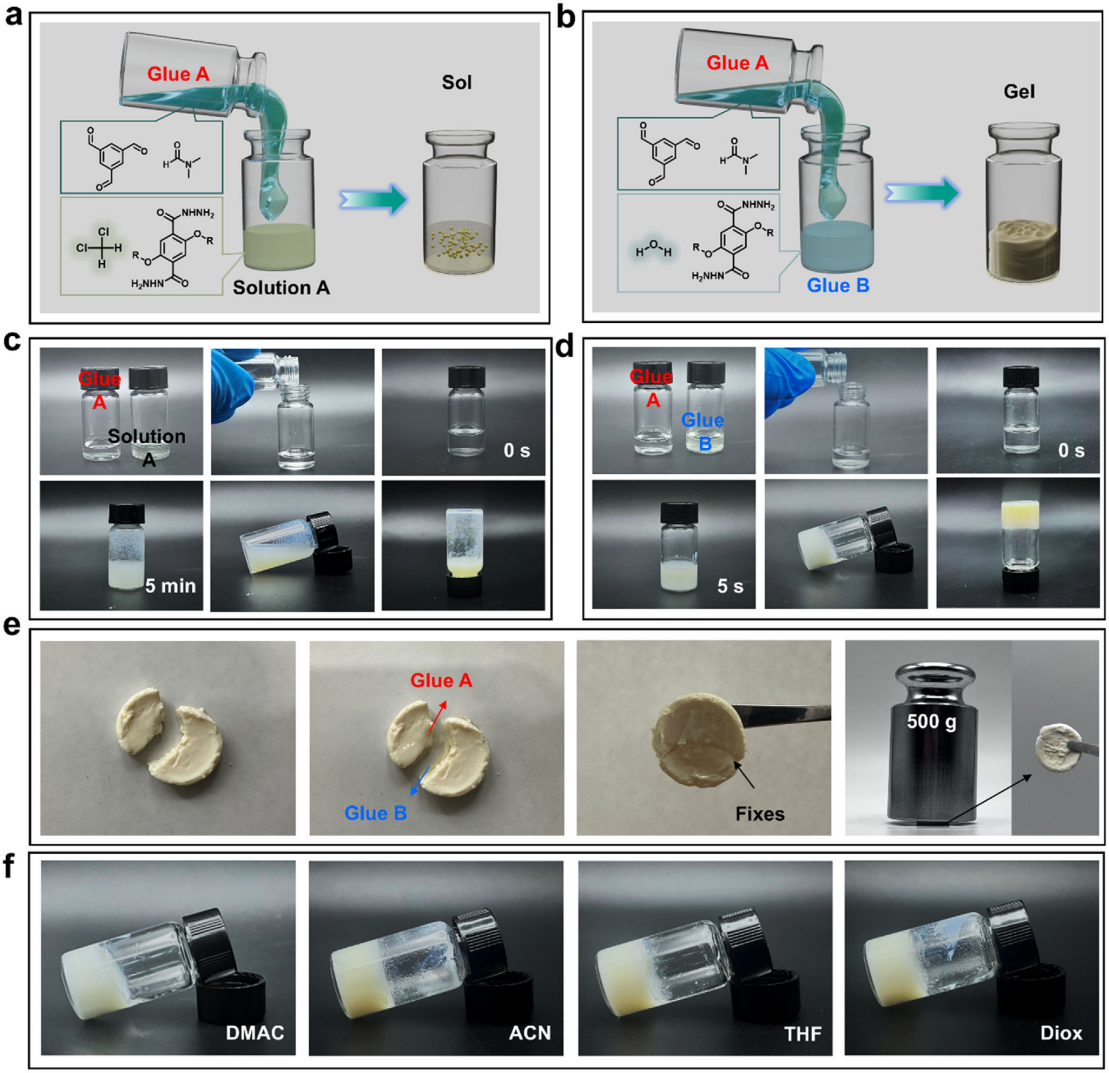
Synthesis of COF‐PEO‐Me. Schematic diagram of a) COF sol preparation and b) gel preparation. c) The images show the preparation of a COF sol by mixing an A gel with an A solution (DCM). d) Images showing the preparation of gel COF by mixing of A&B glues. e) Rapid repair of COF gel blocks by mixing A&B gels and load‐bearing capacity of repaired COF‐PEO‐Me gel. f) Digital images of gel prepared with different solvents.

To emphasize the significance of HBEiMS in COF molding processing, we chose stilbene synthesized via the polymerization reaction of benzene‐1,3,5‐tricarbaldehyde (TF) with 2,5‐bis (2‐methoxyethoxy) terephthalohydrazide (PEO‐Me) linked COFs. The classical Liquid‐Solid Phase Transformation (L‐SPT) method involved in traditional polymer synthesis was a source of inspiration for this work.^[^ ^32^ ^]^ It utilized solvent/non‐solvent exchange of highly concentrated polymer solutions to produce a liquid‐liquid split phase up to gel curing. As shown in **Figure** **1**a,b, we prepared Solution A, Glue A, and Glue B separately in order to avoid aggregation and sedimentation that occurs in the one‐pot method. The synthesis of COFs is remarkably straightforward, requiring only the mixing of the two appropriate solvents (Glue A and Glue B). Experimental observations showed that the mixing of Solution A and Glue A produced COF‐PEO‐Me powder within 5–10 min (Figure 1c, Video S1, Supporting Information). Strong Tyndall effect confirms production of COF sol (Figure S6, Supporting Information). As a comparison, the mixing of Glue A and Glue B reacted rapidly and formed a COF‐PEO‐Me gel within 5 s (Figure 1d, Video S2, Supporting Information). In these two cases, the total amount of monomers, the reaction temperature, and the catalyst remain unchanged. The difference is that hydrogen bonding occurs between the DMF and water used in Glue A & B, respectively. This control experiment highlights the critical role of inter‐solvent hydrogen bonding interactions in COF molding. As illustrated in Figure 1e, Glue A and Glue B successfully repaired the gel during the preparation process. The repairment process also confirms the existence of hydrogen bonding interactions between A&B gels. The restored gel demonstrated the capacity to support up to 500 g of weight in its dry state without sustaining any damage. Comparison of the digital images in Figure 1f and Table S1 (Supporting Information) shows that the change in the form of outputs during COF formation is influenced by the presence or absence of hydrogen bonding interactions between different solvents.

### 2.2 Understanding of the Gel‐Formation Mechanism

**Figure 2:**
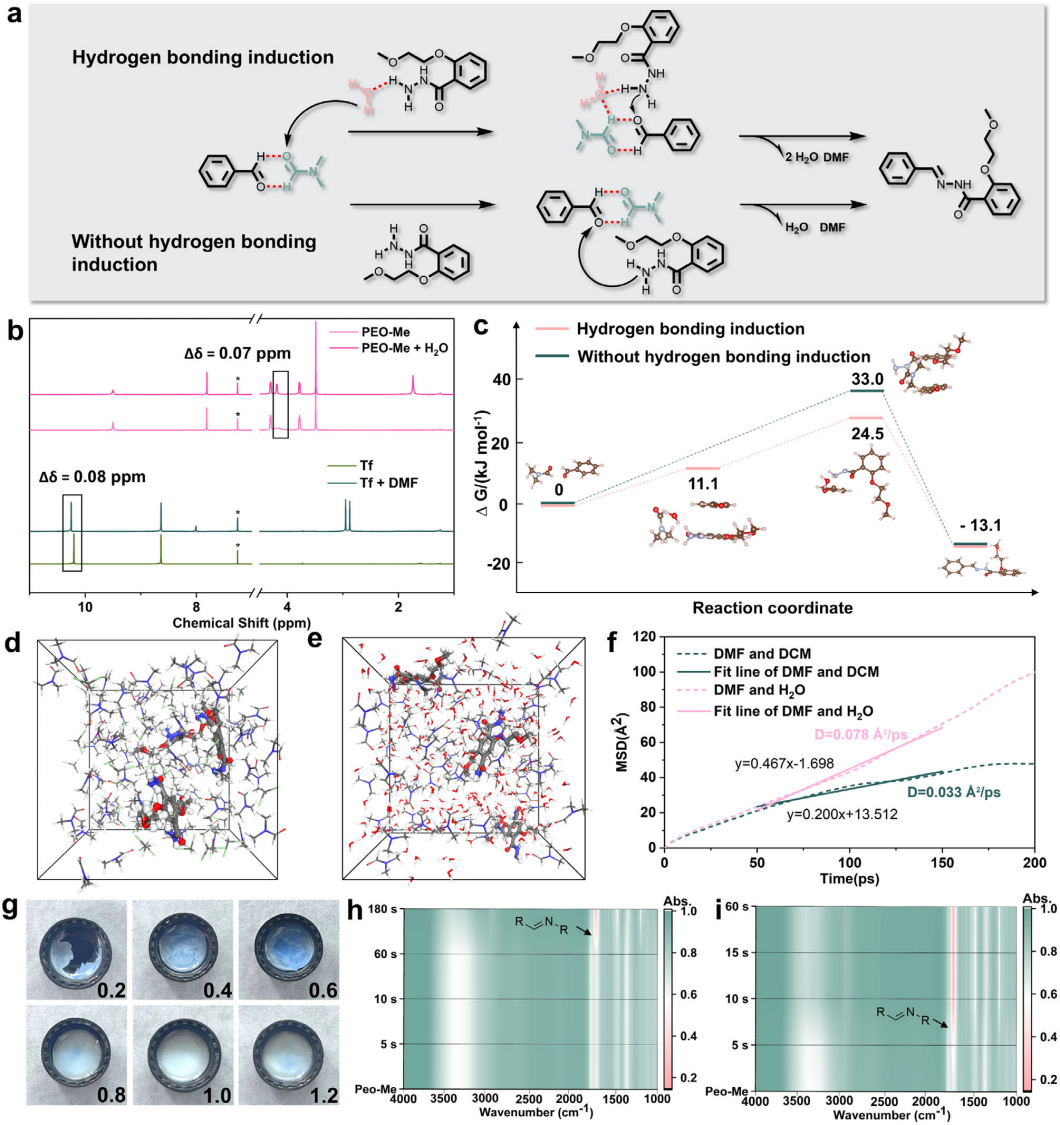
a) Mechanism of covalent self‐assembly driven by solvent hydrogen bonding. b) 1H‐NMR spectra of monomer and hydrogen bonding between monomer and solvent. c) Schematic representation of the free energies for the two reaction paths. Crosslinking models for the construction of covalent organic framework “A&B” gels by hydrogen bond exchange‐induced microphase separation. d) DMF‐DCM model and e) DMF‐H2O model. f) MSD curve. g) COF‐PEO‐Me gels constructed with different water and DMF fractions. In situ infrared spectroscopy of h) sol and i) gel.

The pronounced variations in material properties induced by different solvent compositions necessitate a systematic investigation of their underlying formation mechanisms. The traditional solvothermal method prepares the COF crystalline network through the “self‐correction” mechanism.^[^ ^33^ ^]^ Heterogenous nucleation and uncontrollable mesoscale self‐assembly during the reaction process make the COF output as a powder instead of a gel. In contrast, in the HBE‐regulated self‐assembly strategy, pre‐assembly of intermediate molecules was achieved by hydrogen bonding between DMF and water molecules during mixing, ensuring the correct positioning and orientation of functional groups, which facilitated rapid and efficient covalent polymerization, forming an ordered framework (**Figure** **2**a). Meanwhile, the microphase separation induced by mixing DMF and water molecules realized the homogeneous phase separation of the COF crystalline network at elevated concentration in the reaction system. The hydrogen‐bonding interactions between the solvent molecules and the COF crystalline network further maintained the stability of the generated gel. In order to investigate the solvent hydrogen bonding‐driven pre‐assembly process, bonding interactions between solvent molecules and building blocks. The formation of hydrogen bonds would lead to the chemical shift of functional groups to higher fields in the spectra. It is obvious that the block hydrogen spectra first, we performed NMR titrations to probe the all have a shift of the characteristic peaks towards the upper field after the addition of the corresponding solvent (Figure 2b), which suggests that hydrogen bonds are formed between the solvent molecules and the functional groups of the building blocks. Theoretical calculations were performed to compare the two reaction processes. As shown in Figure 2b, the maximum energy barrier to be overcome for the condensation reaction process without the involvement of solvent hydrogen bonding is 33 kcal mol^−1^, whereas in the solvent hydrogen bonding preassembly method, the energy barriers of 11.1 and 13.4 kcal mol^−1^ are crossed in steps (totaling 24.5 kcal mol^−1^). This comparison suggests that the solvent hydrogen bonding‐modulated self‐assembly strategy is less energetic than the conventional solvothermal method. To explore the function of solvents in this self‐assembly process, the role of hydrogen bonding when mixing A&B gums was further illustrated by modulating the volume ratio of solvents. DMF and water molecules would form different degrees of hydrogen bonding according to different ratios (Figure S6a, Supporting Information). It was confirmed by Raman spectra that the characteristic peak of ‐OH at 3400 cm^−1^ in the Raman spectra gradually decreased with the rise of DMF content in its mixed system with water (Figure S6b,c, Supporting Information). This also indicates that the water molecule formed hydrogen bonds with DMF, destroying the original hydrogen bond nets of water molecules. This exchange of hydrogen bonds further affects the diffusion of the small molecular building blocks of COF in the mixed solvent. Two solution systems were modeled and their properties were analyzed using the amorphous unit module in Material Studio (MSD) to analyze their properties (Figure 2d,e). According to the MSD simulations (Figure 2f), it was shown that the diffusion rate of PEO‐Me in DMF‐H2O was significantly higher than that of DMF‐DCM (from 0.033 to 0.078 Å^2^ per ps) due to the hydrogen bonding between DMF and H2O. The calculations further emphasize that the HBE between DMF and H2O accelerates the monomer diffusion rate, which is consistent with the experimental results shown in Videos S1 and S2 (Supporting Information).

As shown in Figure 2g, the COF‐PEO‐Me gels obtained by mixing A&B gels experienced a trend from being inhomogeneous to being homogeneous and then back to inhomogeneous during the increase of *V*_DMF_:*V*_H2O_ from 0.2 to 1.2. The most homogeneous gels were obtained at equal proportions of DMF and water in the mixture, and this trend was attributed to the varied types of hydrogen bonding between DMF and water molecules at different proportions. The time‐dependent in situ IR spectra showed that the characteristic band of the C═N double bond was observed at 120 s without the involvement of hydrogen bonds (Figure 2h); whereas HBE‐driven self‐assembly shortened the reaction time to 5 s (Figure 2i). This is consistent with the calculation of the energy barriers of the two reaction paths in Figure 2b. Hydrogen bonding interactions between solvents induced covalent self‐assembly of the monomers and stabilized the phase separation of the bulk material after mixing of the A&B gels. To verify the general scope of this strategy, we calculated the electrostatic potentials and possible hydrogen bonding interactions for solvents commonly used in solvothermal reactions (Figure S7, Supporting Information). By mixing the two types of solvents in the same configuration, the prepared systems show consistent experimental phenomena in Table S1 (Supporting Information).

### 2.3 Configuration and Properties of the COF Gel

**Figure 3:**
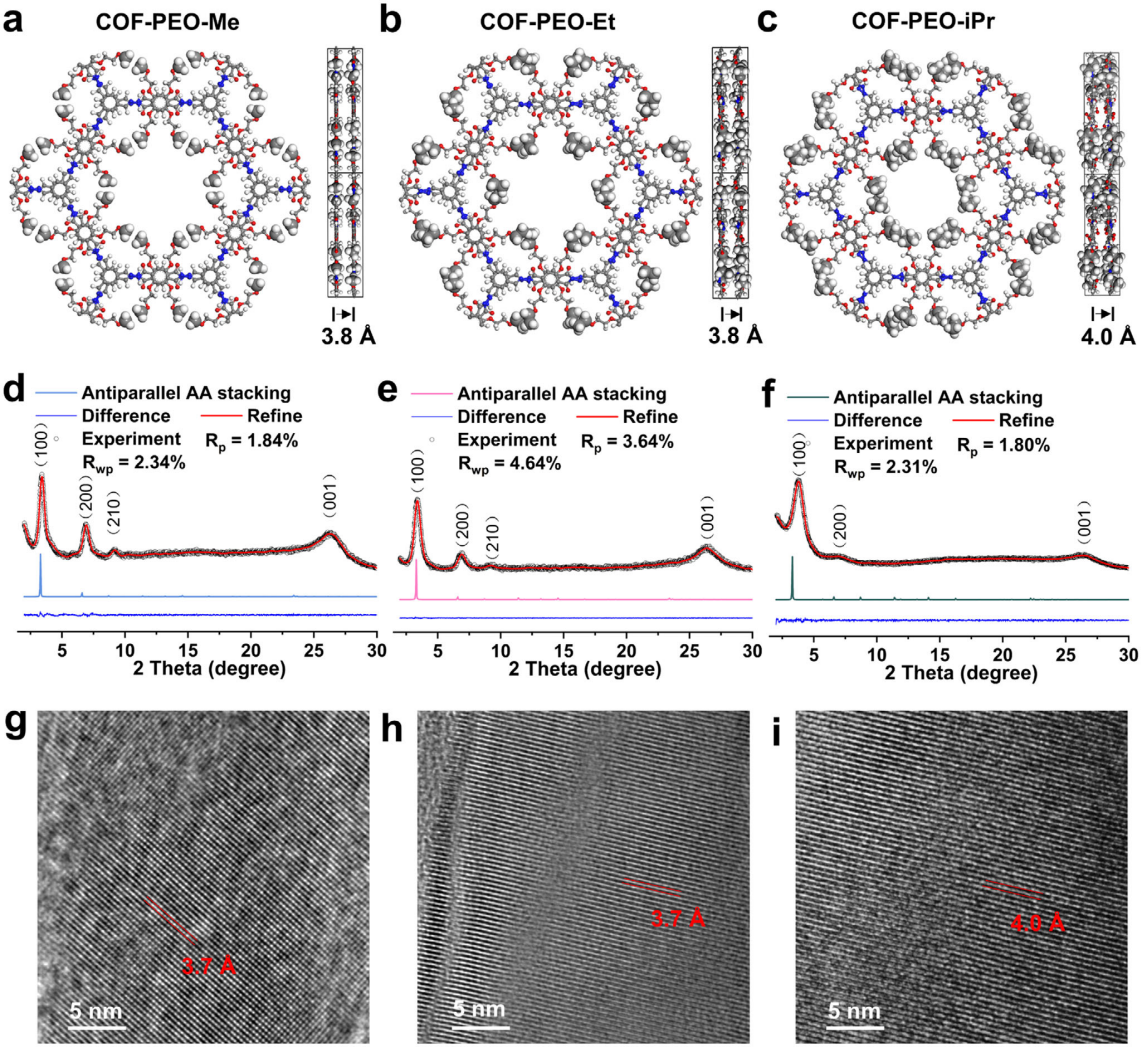
Structures for 3 different COF studied. a) COF‐PEO‐Me, b) COF‐PEO‐Et and c) COF‐PEO‐iPr. PXRD patterns of d) COF‐PEO‐Me, e) COF‐PEO‐Et and f) COF‐PEO‐iPr. TEM images of g) COF‐PEO‐Me, h) COF‐PEO‐Et, and i) COF‐PEO‐iPr.

**Figure 4:**
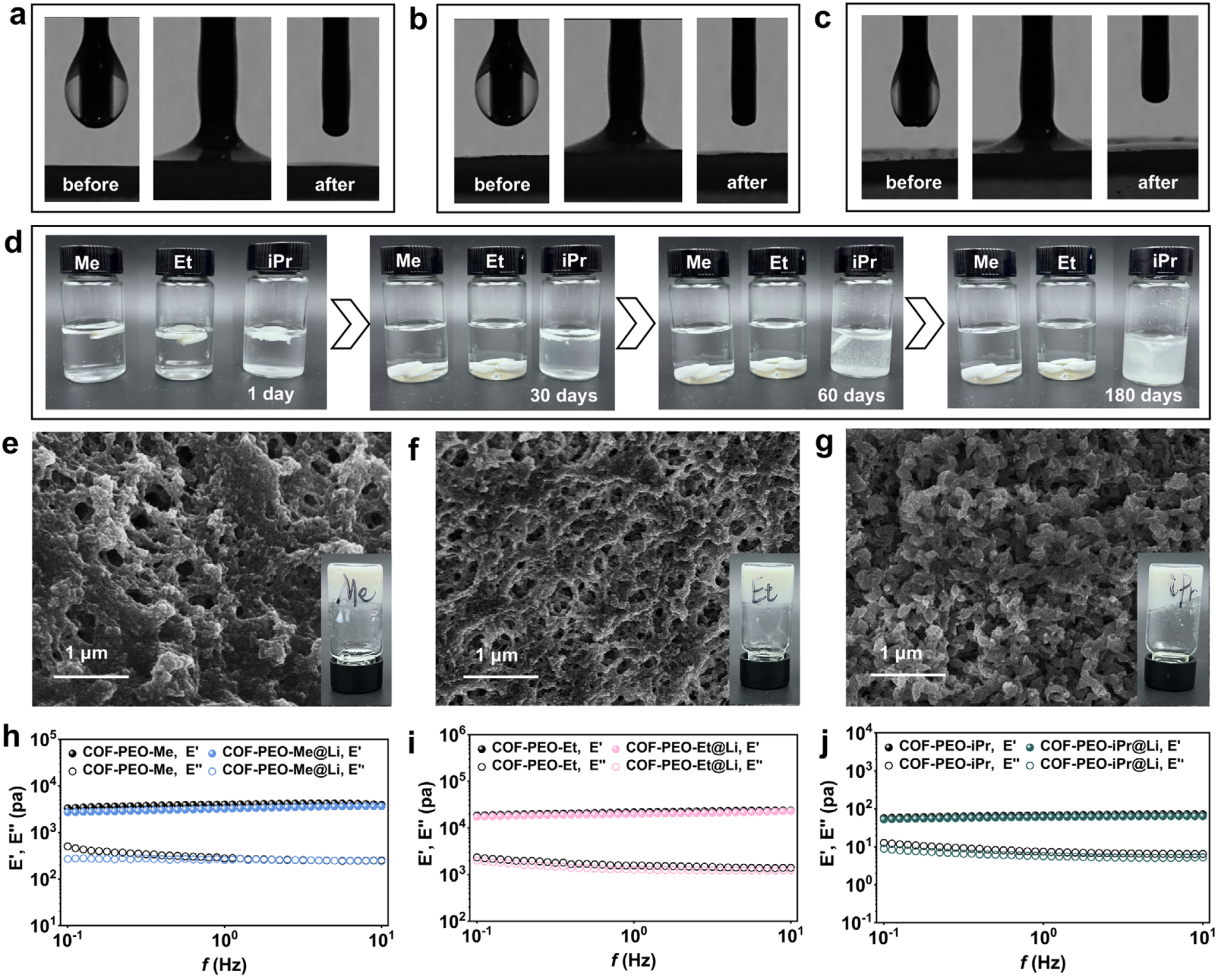
Digital images of contact angle between a) electrolyte and COF‐PEO‐Me, b) COF‐PEO‐Et and c) COF‐PEO‐iPr. Stability testing of d) three COF gels in electrolytes. EDS images of e) COF‐PEO‐Me, f) COF‐PEO‐Et, and g) COF‐PEO‐iPr. Storage modulus (*E*′) (filled circles) and loss modulus (*E*″) (hollow circles) of h) COF‐PEO‐Me, i) COF‐PEO‐Et, and j) COF‐PEO‐iPr.

To modulate the microenvironment of Li^+^ transport within COF channels, we qualitatively modified the end groups of the side chains. Three COF gels, namely COF‐PEO‐Me (**Figure** **3**a), COF‐PEO‐Et (Figure 3b), and COF‐PEO‐iPr (Figure 3c), were successfully synthesized by modifying the terminal groups of the side chains in the structural blocks, using a solvent‐driven self‐assembly strategy based on hydrogen bonding. The structure of the side‐chain‐modified hydrazide unit can limit the rotation of intramolecular bonds through intra‐ and interlayer hydrogen bonding due to its bond dipole moment, which makes the constructed COFs tend to stack antiparallelly. Powder X‐ray diffraction (PXRD) experiments showed strong diffraction peaks for all three COF gels, indicating high crystallinity. The PXRD patterns of COF‐PEO‐Me showed distinct diffraction peaks at 2*θ* values of ≈3.4, 6.9 and 9.1° (Figure 3d).^[^ ^34^ ^]^ They correspond to the simulated antiparallel AA stacking results for the 100, 200, and 210 reflectors. The PXRD patterns of COF‐PEO‐Et and COF‐PEO‐iPr exhibit corresponding (100) plane angles of 3.3° (Figure 3e) and 3.7° (Figure 3f), respectively. The broad peaks around 25° in the plots are all attributed to interlayer π–π stacking. The difference in lattice size is evidenced by variations in their stacking patterns, suggesting unique spatial configurations (Figures S8–S10, Supporting Information). Distinguishing the conventional AA and AB models, intra‐ and interlayer hydrogen bonding restricts intramolecular bond rotation leading to a 0.2 Å reduction in the interlayer π–π stacking of antiparallel AA stacks. High‐resolution transmission electron microscopy (HRTEM) images revealed distinct crystal structures, with lattice spacing of 3.7 Å for COF‐PEO‐Me and COF‐PEO‐Et (Figure 3g,h), and 4.0 Å for COF‐PEO‐iPr, respectively (Figure 3i). These results were consistent with the layer spacing observed for the antiparallel AA stacks. The new emerged characteristic band at 1645 cm^−1^ (C═N) in the Fourier transform infrared (FTIR) spectroscopy confirms the successful preparation of COF‐PEO‐Me/Et/iPr, as shown in Figure S11 (Supporting Information). Given the potential of the three materials for energy storage applications, it is essential to conduct an in‐depth analysis of their suitability and stability with electrolytes. The initial assessment of the gel materials' affinity to the electrolyte was performed using contact angle measurements. As shown in **Figure** **4**a–c. COF‐PEO‐Me/Et/iPr demonstrate a strong affinity with the electrolyte, providing a solid basis for subsequent electrolyte replacement.

To assess the stability of the three gels, we immersed them in an electrolyte solution. COF‐PEO‐Me and COF‐PEO‐Et appeared stable for up to 6 months, whereas COF‐PEO‐iPr disintegrated within 1 month (Figure 4d). The IR spectra indicated that the C═N band at 1645 cm^−1^ remained intact (Figure S11, Supporting Information), suggesting that the gel's disintegration was merely due to the dispersion of the gelatinous mass, without any cleavage of chemical bonds. Additionally, scanning electron microscopy (SEM) analysis provided insights into the differences in the stability of the three gels. The SEM images of COF‐PEO‐Me and COF‐PEO‐Et displayed distinct reticular entanglements (Figure 4e,f), with visible pores in the gel's secondary structure. In contrast, the SEM image of COF‐PEO‐iPr revealed its block‐like structures formed by interconnected, coral‐like particles (Figure 4g). Rheological analysis revealed that the three gel materials exhibit frequency‐independent mechanical stiffness, with the stored Young's modulus (E’) of 3.39 kPa for COF‐PEO‐Me (Figure 4h), 18.53 kPa for COF‐PEO‐Et (Figure 4i), and 0.05 kPa for COF‐PEO‐iPr (Figure 4j). It is significant that the storage modulus (*E*′) of all three gel materials was substantially higher than their loss modulus (*E*″), indicating gel‐like mechanical properties. Additionally, the rheological characteristics of our COF gels remained unchanged after electrolyte replacement tests. Additionally, SEM elemental analysis confirmed that the F and S elements in the LiTFSI, after electrolyte replacement, were evenly distributed throughout the material (Figure S14, Supporting Information). Laser confocal scanning of the surface of the gel block loaded into the button cell revealed that the overall surface roughness was only at the micron scale (Figure S15, Supporting Information), which provides a solid foundation for the subsequent assembly of the gel electrolyte into components.

### 2.4 Electrochemistry Properties of the COF Gel

**Figure 5:**
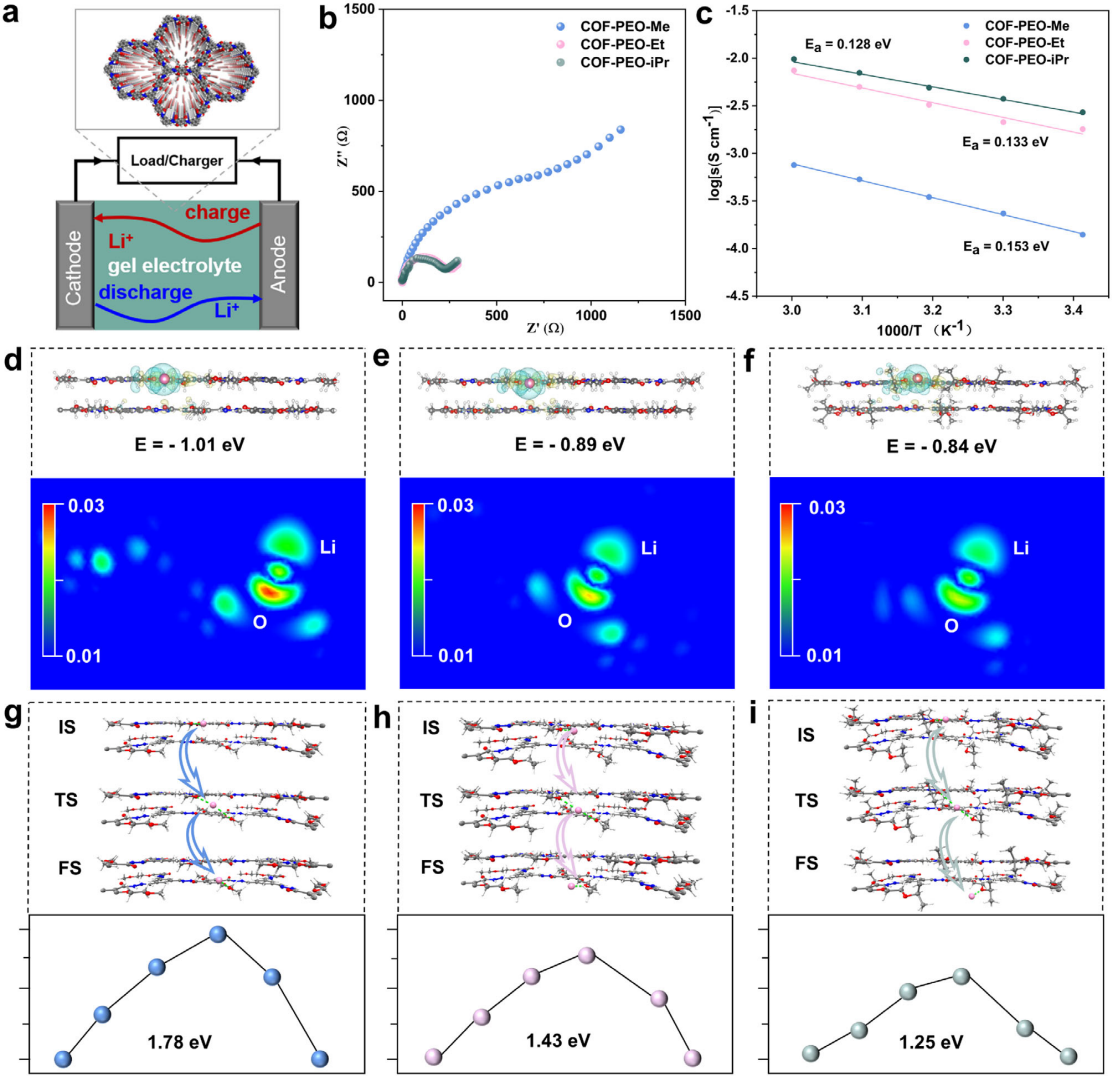
a) Testing cell for ionic conductivity measurement. b) Nyquist curves of three gel electrolytes at room temperature. c) Arrhenius ionic conductivity plots and activation energies for three COF gels. Binding energy (top) and electron transfer (bottom) of Li+ to side‐chain oxygen atoms for d) COF‐PEO‐Me, e) COF‐PEO‐Et and f) COF‐PEO‐iPr. Axial migration pathways of Li+ in g) COF‐PEO‐Me, h) COF‐PEO‐Et, and i) COF‐PEO‐iPr.

The ionic conductivities of the three gel‐assembled symmetric cells were assessed using electrochemical impedance spectroscopy (EIS) over a temperature range of 20 to 80 °C (**Figure** **5**a). At 20 °C, as illustrated in Figure 5b, COF‐PEO‐Me demonstrated an ionic conductivity of 0.14 mS cm^−1^. In contrast, COF‐PEO‐Et and COF‐PEO‐iPr showed a remarkable enhancement in ionic conductivity, with values increasing by an order of magnitude to 1.8 and 2.8 mS cm^−1^. This improvement can be attributed to the strategic modifications in the side chain end groups. Furthermore, the activation energy values of these gel electrolytes, calculated using Arrhenius' law, were found to be relatively low. The values were 0.153 eV for COF‐PEO‐Me, 0.133 eV for COF‐PEO‐Et, and 0.128 eV for COF‐PEO‐iPr, as depicted in Figure 5c. To gain deeper mechanistic insights, the host–guest adsorption model was employed to simulate the interaction structure between lithium ions and the framework. Through structural optimization, the binding energy between the lithium ion and the oxygen atom on the side chain in COF‐PEO‐Me was calculated to be 1.01 eV, as shown in Figure 5d (top). Upon modifying the side‐chain end groups from methyl to ethyl and isopropyl within the COF pores, the binding energies between the lithium ions and the oxygen atoms on the side chains decreased to 0.89 eV and 0.84 eV, respectively (Figure 5e,f, top). This reduction in binding energy is primarily due to the electron‐donating effect of the neighboring methyl group, which allows the oxygen atom to accumulate more electron density (Figure 5d, bottom). Conversely, the ethyl and isopropyl groups drop the electron distribution around the oxygen atom, thereby weakening the binding interaction (Figure 5e,f, bottom). Consequently, lithium ions exhibit weaker binding to the side chains in the pores of COF‐PEO‐Et and COF‐PEO‐iPr, which contributes to the observed enhancement in ionic conductivity. The migration barriers of Li+ ions perpendicular to the COF‐PEO‐Me/Et/iPr pores were investigated, as shown in Figure 5g,h. For COF‐PEO‐Me, the migration barrier was determined to be 1.78 eV. This barrier decreased to 1.43 eV for COF‐PEO‐Et and further to 1.25 eV for COF‐PEO‐iPr. These results indicate that the interaction between lithium ions and the side chains is significantly weaker in the pores of COF‐PEO‐Et and COF‐PEO‐iPr, facilitating easier migration of lithium ions and resulting in higher ionic conductivity.

### 2.5 Cycling Performance of Cells

**Figure 6:**
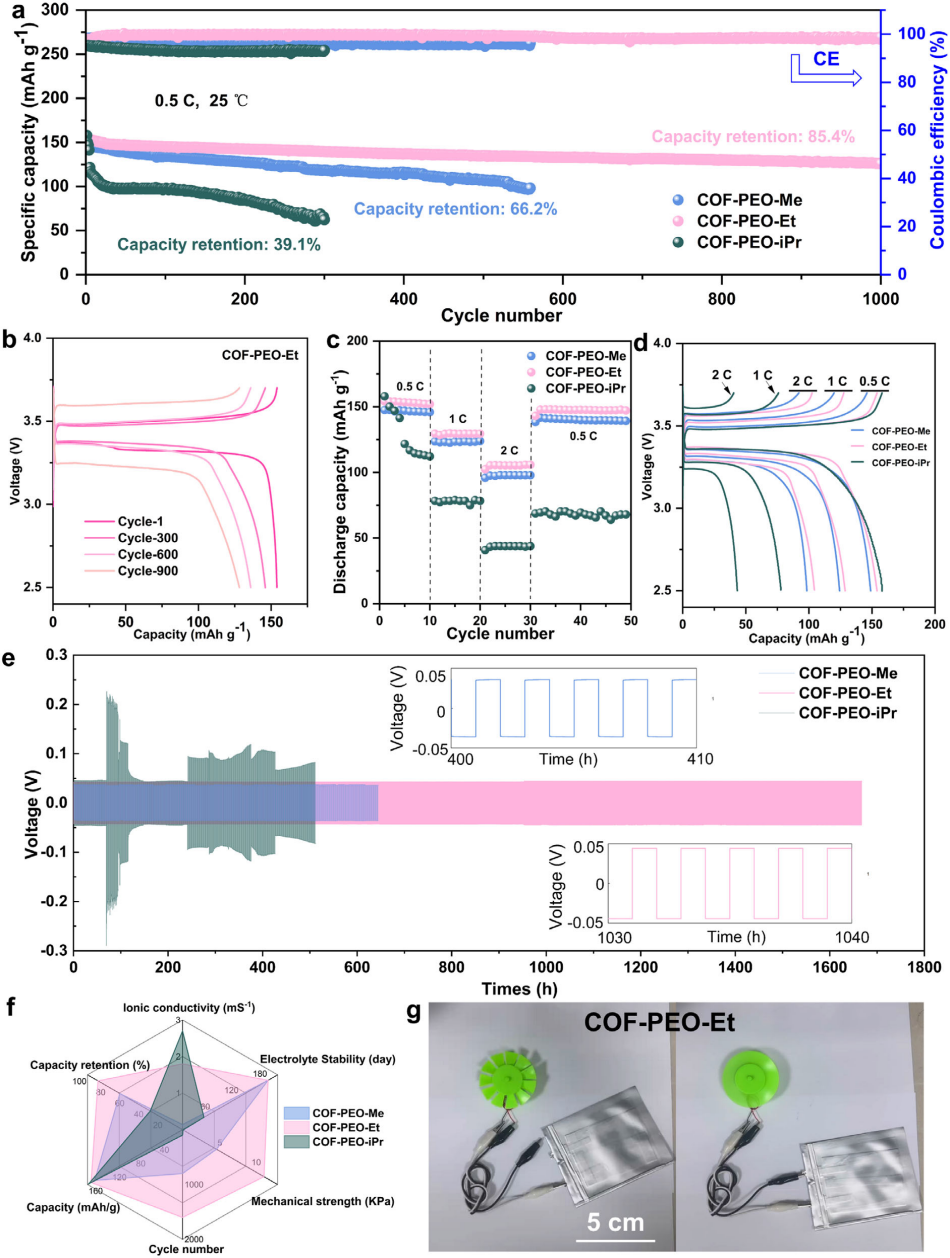
a) Cycling performances of LiFePO4/gel/Li cells at 0.5 C. b) Discharge–charge profiles for COF‐PEO‐Et; c) Cycling performances of LiFePO4/gel/Li cells at 0.5, 1, and 2 C. d) Discharge–charge profiles for COF‐PEO‐Et. e) Discharge–charge profiles for three COF gels at 0.5, 1, and 2 C. e) Long‐term cycling of batteries with three COF gels (Li/Li). f) Comparison of the integrated electrochemical properties of three COF gels. g) Schematic diagram of soft–pack battery and its driving fan assembled by COF‐PEO‐Et gel.

To validate the feasibility of gel electrolytes in battery, we conducted comprehensive electrochemical evaluations on Li | Gel | LFP button cells incorporating COF‐PEO‐Me/Et/iPr gel electrolytes at ambient temperature. The cycling performance analysis, as depicted in **Figure** **6**a, reveals distinct capacity retention characteristics among the different gel formulations. The COF‐PEO‐Me based cell exhibited a capacity retention of 66.2% after 580 cycles at 0.5 C. In contrast, the COF‐PEO‐Et based cell demonstrated superior cycling stability, maintaining 85.4% of its initial capacity after 1000 cycles, with an exceptionally low capacity decay rate of 0.015% per cycle (Figure 6b). While the COF‐PEO‐iPr based cell initially displayed the highest specific capacity, it suffered from rapid capacity degradation, retaining only 39.1% of its initial capacity after 300 cycles. The rate performance assessment further highlights the electrochemical superiority of the COF‐PEO‐Et based cells (Figure 6c), which delivered reversible capacities of 154, 129, and 105 mAh g^−1^ at current densities of 0.5, 1, and 2 C, respectively (1 C = 170 mAh g^−1^). The COF‐PEO‐iPr based cells exhibited a linear capacity decline from 158 to 78 mAh g^−1^ when the current density was increased from 0.5 C to 1 C. More importantly, this capacity loss was irreversible, as evidenced by the sustained capacity of only 70 mAh g^−1^ when the current density was returned to 0.5 C (Figure 6d).

To further investigate the electrochemical stability of these gel electrolytes, Li/Gel/Li symmetric cells were fabricated and subjected to long‐term cycling tests (Figure 6e). The COF‐PEO‐Et based symmetric cell demonstrated exceptional cycling stability, maintaining stable operation for over 1600 h at an area capacity of 1 mAh cm^−2^. In comparison, the COF‐PEO‐Me and COF‐PEO‐iPr based cells showed significantly shorter lifespans of 600 and 80 h, respectively. This performance disparity can be primarily attributed to variations in ionic conductivity and electrochemical stability among the different gel formulations (Figure 6f). Scanning electron microscopy observations revealed that after cycling, little‐to‐no lithium dendrite growth was observed on the lithium metal surface of COF‐PEO‐Me/Et, while the lithium metal surface of COF‐PEO‐iPr exhibited noticeable lithium dendrites (Figure S19, Supporting Information). The superior mechanical properties of COF‐PEO‐Et enabled the successful fabrication of a large‐scale, flexible pouch cell capable of powering up an electric fan at room temperature (Figure 6g). The safety characteristics of these gel electrolytes was evaluated through combustion tests, revealing remarkable flame‐retardant properties (Figure S20, Supporting Information). This enhanced safety profile is likely due to the presence of nitrogen‐rich frameworks in the COF‐PEO structure, which effectively disrupt the chain reactions involved in electrolyte combustion. The comprehensive evaluation of these gel electrolytes demonstrates that COF‐PEO based systems, particularly the COF‐PEO‐Et formulation, offer a promising combination of stable electrochemical performance, mechanical flexibility, and enhanced safety features, making them attractive candidates for next‐generation energy storage applications.

## 3 Conclusion

This study proposed a hydrogen bond exchange‐induced microphase separation strategy for fabrication of hydrazone‐linked covalent organic framework (COF) “A&B” gel electrolytes. The partitioned Glue A and Glue B effectively circumvented the issues of aggregation and sedimentation of powders, which commonly arise from non‐homogeneous nucleation during monomer condensation in conventional one‐pot synthesis methods. Through hydrogen bonding exchange‐induced phase transition, we achieved homogeneous separation of bulk materials, enabling the successful preparation of highly crystalline COF gels within an unprecedented short period of 5 s at ambient temperature. Furthermore, charge transport was regulated through oxygen atoms within the COF pores via strategic modification of terminal groups on the monomer side chains. This approach allowed for meticulous control over the binding strength of Li^+^ at oxygen sites, ultimately facilitating the development of gel electrolytes with exceptional cycling stability for up to 1600 h in long‐term cycling tests. The demonstrated rapid and energy‐efficient gel preparation methodology establishes a groundbreaking pathway for possible industrialization and practical application of COF materials.

## Conflict of Interest

The authors declare no conflict of interest.

## Author Contributions

Z.F.: material preparation and structural analysis, data analysis, writing‐first draft and editing. Z.L.: conceptualization of works. F.Z.: Data organization. Y.H.: conceptualization of works. C.P.: conceptualization of works. S.G.: resources and supervision. J.T.: article modification. B.W.: data organization and article modification. J.Y.: resources and visualization. G.Y.: conceptualization of works, funding, resources, supervision, review, writing and editing.

## Supporting information



Supporting Information

Supplemental Video 1

Supplemental Video 2

Supplemental Video 3

## Data Availability

The data that support the findings of this study are available from the corresponding author upon reasonable request.
