# Supplementary material for: Establishing Covalent Organic Framework “A&B” Gel via Hydrogen Bond Exchange‐Induced Microphase Separation
Source: Adv Sci (Weinh). 2025 Aug 19;12(42):e08484. doi: 10.1002/advs.202508484 (PMC12622503; doi:10.1002/advs.202508484)
Supplement: Supplementary file 1 — Supporting Information [file ADVS-12-e08484-s002.docx]

**Supporting information**

**Construction of Covalent Organic Framework "A&B" Gel via Hydrogen Bond Exchange-Induced Microphase Separation**

Zhiwen Fan ^[a]^, Zihao Liao ^[a]^, Feng Zhang ^[a]^, Yang Huang ^[b]^, Chunyue Pan ^[a]^, Shuai Gu ^[a]^, Juntao Tang ^[a]^, Baosheng Wei ^[a]^, Jiayin Yuan * ^[c]^, Guipeng Yu * ^[a]^

[a] Z. Fan, Z. Liao, F. Zhang, Y. Huang, C. Pan, S. Gu, J. Tang, B. Wei, Prof. G. Yu
College of Chemistry and Chemical Engineering, Central South University, 410083 Changsha, Hunan, P. R. China
E-mail: gilbertyu@csu.edu.cn

[b] Y. Huang
Shenzhen Cham new energy Battery Technology Co., 523000 Dongguan, China

[c] Prof. J. Yuan
Department of Materials and Environmental Chemistry, Stockholm University, SE-10691 Stockholm, Sweden.
E-mail: Jiayin.yuan@mmk.su.se

Table of Contents

[1. General information 3](#_Toc143120199)

[2 Synthesis of monomers 5](#_Toc143120200)

[3. Synthesis of COF materials 9](#_Toc143120201)

[4. Characterization of monomer and COF materials 15](#_Toc143120202)

[5. Reference 34](#_Toc143120202)

# 1. General information

***Materials***

Benzene-1,3,5-tricarbaldehyde (TF), dimethyl 2,5-dihydroxyterephthalate, hydrazinium hydroxide, *p*-toluenesulfonyl chloride, ethylene glycol monomethyl ether, 2-ethoxyethanol and 2-isopropoxyethanol were purchased from Energy Chemical. Ethanol, potassium Carbonate (K_2_CO_3_), sodium hydroxide (NaOH), dimethylacetamide, tetrahydrofuran, acetonitrile, *N, N*-dimethylformamide, dimethyl sulfoxide, trichloromethane, acetone, and dichloromethane were supplied by Energy Chemical. All the chemicals were used directly without further purification.

***Instrumentation***

**Solution-Phase Nuclear Magnetic Resonance (NMR)** spectra (^1^H and ^13^C) were recorded on Bruker FT-NMR spectrometer (400 MHz and 100 MHz, Bruker).

**Powder X-ray diffraction (PXRD)** patterns were recorded on Rigaku Mini Flex II diffractometer with Cu Kα line focused radiation working at 40 kV with a current at 40 mA. The scans ranged from 2θ = 2° to 30° with a step size set at 0.03° by Bragg-Brentano. The powder samples were added to the glass and compacted before conducting the measurement.

**Fourier Transform Infrared Spectroscopy (FTIR)** of the monomers and prepared polymers were characterized on a Thermo Fisher Nicolet iS5 spectrometer in a range of 400-4000 cm^-1^ with a resolution of 4 cm^-1^.

**Scanning Electron Microscope (SEM)** was used to characterize the morphology of the samples, which was recorded by a scanning electron microscope (SEM, SU8020 (Hitachi, Japan)).

**Rheometer** analyses were performed using a stress-controlled MCR 302 (Anton Paar, Austria) rheometer to acquire the rheology data of the gels. Sample measurement was conducted in compressed mode frequency scanning, strain amplitude of 0.8%.

**High-resolution transmission electron microscopy (HRTEM)** images were characterized on JEOL/JEM-F200 Electron Microscope at an operating voltage of 200 kV.

***Electrochemical Characterization***

All electrochemical measurements were investigated via coin cells in an MBRAUN glove box under high-purity argon (<0.5 ppm H_2_O and < 0.5 ppm O_2_). Electrochemical impedance spectroscopy (EIS) was tested by assembling stainless steel (SS)| CGE |SS cells from 0.1 Hz to 100 kHz with an amplitude of 10 mV via Biological SP-200. The ionic conductivity (σ) was calculated according to Equation (1), in which L is S as the effective contacting area among electrolytes and SS, R presents the resistance value of the bulk electrolyte, and L is the thickness of the polymer electrolytes.


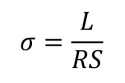
 (1)

Arrhenius plots were obtained by calculating ionic conductivity at a temperature range of 25-65 ℃ via Biological SP-200. A is the conductivity pre-exponential factor, Ea is the activation energy, and T is the absolute temperature, R is the ideal gas constant. The

computational formula of the Ea: the Ea of polymer electrolytes was calculated by Arrhenius relation from Equation (2).


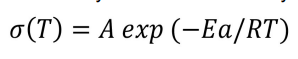
 (2)

The cycling stability of Li| CGE |Li cells was conducted using the battery testing system (LAND CT2003) at 25 ℃. The full cells were assembled with composite cathodes, polymer electrolytes, and lithium metal anodes via ordinal stacking. The cycle performance of Li| CGE |LFP cell is tested between 2.5 and 3.7 V (vs Li+/Li) at current densities of 0.5 C for long cycle testing, and 0.5 C, 1.0 C, and 2 C (1 C = 170 mAh g-1) for rate testing.

***Computational calculations***

Geometry optimizations are performed with the Perdew–Burke–Ernzerhof (PBE)^1^ exchange-correlation functional. The dispersion interaction is corrected by the Grimme method (DFT-D3), which yields accurate absorption heights and energies for a variety of atoms.^2^ The plane-wave cutoff was set at 400 eV. To avoid interaction between periodic images, a 15 Å vacuum space was included in the xyz-direction to avoid interaction between periodic images. For Brillouin zone integration, the Γ-point is considered in the geometry optimizations while otherwise a 4 × 4 × 1 Monkhorst-Pack k-grid is used.^3^ Gaussian smearing of 0.05 eV is adopted. An energy convergence criterion of 10^−6^ eV and a Hellmann–Feynman force criterion of 0.02 eV Å^−1^ are adopted.

Ea = Etotal − (Esubstrate + Especies)

The binding energy between COF monolayers is calculated as E_b_ = E_bilayer_ − 2E_monolayer_, with E_monolayer_ being the total energy of the COF monolayer and E_bilayer_ being the total energy of the COF bilayer.

All quantum-chemical calculations were performed with the Vienna Ab initio Simulation Package (VASP) ^4,5^ code.

***Batteries Assembly***

Due to the sensitivity of lithium metal and lithium salts to air, all batteries were assembled into a glove box filled with argon (O_2_ and H_2_O < 0.1 ppm), and different electrolytes were used for assembly according to test requirements.

(1) Preparation of LiFePO_4_ electrodes: First, the LiFePO_4_ as electrode active material, binder PVDF and conductive additive Super P were mixed according to the mass ratio of 80:10:10. Next, the NMP solvent was added and the mixture was stirred to a homogeneous state, before it was coated on the aluminum foil collector. Then, it was placed in a vacuum drying oven at 60 ℃ for 24 h. The aluminum foil collector was then dried in a vacuum drying oven at 60 ℃.

(2) Preparation of COF-PEO-Me/Et/iPr gel electrolyte: the prepared COF-PEO-Me/Et/iPr gel was cut into discs with a radius of 5 mm using a mold, which was subsequently replaced with a commercial electrolyte for backup.

(3) LiFePO_4_ | COF-PEO-Me/Et/iPr | Li and Li | COF-PEO-Me/Et/iPr | Li Button Cell Assembly Battery Assembly: In assembling the LiFePO_4_ | COF-PEO-Me/Et/iPr | Li button cell, LiFePO_4_ electrodes and lithium metal were used as the positive electrodes and negative electrodes, respectively. The battery was assembled by using COF-PEO-Me/Et/iPr. After encapsulation, the cells were shelved at room temperature for 12 h to ensure sufficient wetting of the electrolyte to the electrodes. Finally, electrochemical performance tests were performed. Li | COF-PEO-Me/Et/iPr | Li button cell was assembled with lithium metal for both positive and negative electrodes, and the rest of the steps was the same as above. The assembly of soft-pack batteries is similar to that of button batteries, with the positive and negative electrodes and gel electrolyte cut into 5 cm x 5 cm models.

# 2 Synthesis of monomers

**2.1 Synthesis of TEG-Ots-Me/Et/iPr**

TEG-Ots- Me/Et/iPr was synthesized according to the published procedure ^6^.

**2.2 Synthesis of 2,5-bis (2-methoxyethoxy) terephthalohydrazide (PEO-Me)**

2,5-Dihydroxy-1,4-benzenedicarboxylic acid dimethyl ester (1.7 g 7.5 mmol) was dissolved in of DMF (68 mL) in a 250 mL round bottom flask. Then potassium carbonate (2.1 g, 16.2 mmol) and TEG-Ots-Me (3.7 g, 16.2 mmol) were added. The reaction mixture was stirred overnight at 110 °C. After the reaction time, the mixture was hot-filtered and evaporated to dryness. The residue was extracted with dichloromethane and dried over anhydrous sodium sulfate and evaporated to dryness to obtain a crude product as a yellow oil (**1a**).

Dimethyl 2,5-bis (2-methoxyethoxy) terephthalate (**1a**) (1.3 g, 3.8 mmol) was dissolved in 37 mL of ethanol in a 250 mL round bottom flask. Then 5.7 mL (115 mmol) of hydrazine hydrate was added. The reaction mixture was refluxed by stirring at 90 °C for 12 hours. After cooling to room temperature mixture evaporated to dryness. The residue was suspended in water 30 mL and extracted with 3 × 50 mL dichloromethane. The combined organic layers were washed with brine and water and dried over anhydrous sodium sulfate and evaporated to dryness to obtain a crude product and purified with column chromatography (5% methanol in DCM to afford the product as an off white solid (**PEO-Me**). (1.3 g, 82%) ^1^H NMR (400 MHz, Chloroform-*d*): δ = 9.48 (s, 1H), 7.79 (s, 1H), 4.28 (dd, *J* = 5.5, 3.5 Hz, 2H), 3.76 (dd, *J* = 5.4, 3.6 Hz, 2H), 3.47 (s, 3H). ^13^C NMR: δ = 164.79, 151.04, 124.20, 117.00, 70.29, 69.11, 59.12, 20.87.

**2.3 Synthesis of 2,5-bis (2-ethoxyethoxy) terephthalohydrazide (PEO-Et)**

2,5-Dihydroxy-1,4-benzenedicarboxylic acid dimethyl ester (1.7 g 7.5 mmol) was dissolved in of DMF (68 mL) in a 250 mL round bottom flask. Then potassium carbonate (2.1 g, 16.2 mmol) and TEG-Ots-Et (3.9 g, 16.2 mmol) were added. The reaction mixture was stirred overnight at 110 °C. After the reaction time, the mixture was hot-filtered and evaporated to dryness. The residue was extracted with dichloromethane and dried over anhydrous sodium sulfate and evaporated to dryness to obtain a crude product as a yellow oil (**2a**).

Dimethyl 2,5-bis (2-ethoxyethoxy) terephthalate (**2a**) (1.4 g, 3.8 mmol) was dissolved in 37 mL of ethanol in a 250 mL round bottom flask. Then 5.7 mL (115 mmol) of hydrazine hydrate was added. The reaction mixture was refluxed by stirring at 90 °C for 12 hours. After cooling to room temperature mixture evaporated to dryness. The residue was suspended in water 30 mL and extracted with 3 × 50 mL dichloromethane. The combined organic layers were washed with brine and water and dried over anhydrous sodium sulfate and evaporated to dryness to obtain a crude product and purified with column chromatography (5% methanol in DCM to afford the product as an off white solid (**PEO-Et**). (1.2 g, 79%) ^1^H NMR (400 MHz, Chloroform-*d*): δ = 9.47 (s, 1H), 7.74 (s, 1H), 4.27 – 4.21 (m, 2H), 3.78 – 3.72 (m, 2H), 3.57 (q, *J* = 7.0 Hz, 2H), 1.23 (d, *J* = 7.0 Hz, 3H). ^13^C NMR: δ = 164.83, 150.97, 124.13, 116.83, 69.14, 68.07, 66.80, 15.03.

**2.4 Synthesis of 2,5-bis (2-isopropoxyethoxy) terephthalohydrazide (PEO-iPr)**

2,5-Ddihydroxy-1,4-benzenedicarboxylic acid dimethyl ester (1.7 g 7.5 mmol) was dissolved in of DMF (68 mL) in a 250 mL round bottom flask. Then potassium carbonate (2.1 g, 16.2 mmol) and TEG-Ots-iPr (4.1 g, 16.2 mmol) were added. The reaction mixture was stirred overnight at 110 °C. After the reaction time, the mixture was hot-filtered and evaporated to dryness. The residue was extracted with dichloromethane and dried over anhydrous sodium sulfate and evaporated to dryness to obtain a crude product as a yellow oil (**3a**).

Dimethyl 2,5-bis (2-isopropoxyethoxy) terephthalate (**3a**) (1.5 g, 3.85 mmol) was dissolved in 37 mL of ethanol in a 250 mL round bottom flask. Then 5.7 mL (115 mmol) of hydrazine hydrate was added. The reaction mixture was refluxed by stirring at 90 °C for 12 hours. After cooling to room temperature mixture evaporated to dryness. The residue was suspended in water 30 mL and extracted with 3 × 50 mL dichloromethane. The combined organic layers were washed with brine and water and dried over anhydrous sodium sulfate and evaporated to dryness to obtain a crude product and purified with column chromatography (5% methanol in DCM to afford the product as an off white solid (**PEO-iPr**). (0.9 g, 61%) ^1^H NMR (400 MHz, Chloroform-*d*): δ = 9.48 (s, 1H), 7.76 (s, 1H), 4.25 (dd, *J* = 5.5, 3.7 Hz, 2H), 3.79 – 3.74 (m, 2H), 3.67 – 3.63 (m, 1H), 1.21 (d, *J* = 6.1 Hz, 6H). ^13^C NMR: δ = 164.96, 150.96, 124.04, 116.70, 72.18, 69.35, 65.58, 21.92, 18.37.

**2.5 Synthesis of 2,5-bis (2-methoxyethoxy)-N1, N4-di ((E)-styryl) terephthalamide (Model 1)**

Benzaldehyde (100 mg,1.0 mmol) was dissolved in 2 mL of DMF to obtain solution A; PEO-Me (170 mg,0.5 mmol) was dissolved in 2 mL of a 3 M aqueous solution of HOAc to obtain solution B; solution A & B were mixed and then remove the solvent. The white powder (Model 1) was obtained after wet washing and drying with petroleum ether and water. ^1^H NMR (400 MHz, Chloroform-*d*): δ = 11.46 (s, 1H), 8.14 (s, 1H), 7.95 (s, 1H), 7.85 – 7.73 (m, 2H), 7.49 – 7.34 (m, 3H), 4.46 – 4.32 (m, 2H), 3.97 – 3.81 (m, 2H), 3.50 (s, 3H). ^13^C NMR: δ = 160.66, 150.87, 149.01, 133.91, 130.48, 128.69, 127.82, 124.94, 117.34, 70.35, 68.63, 58.59, 18.41.

# 3. Synthesis of COF A&B Gel and COF Sol


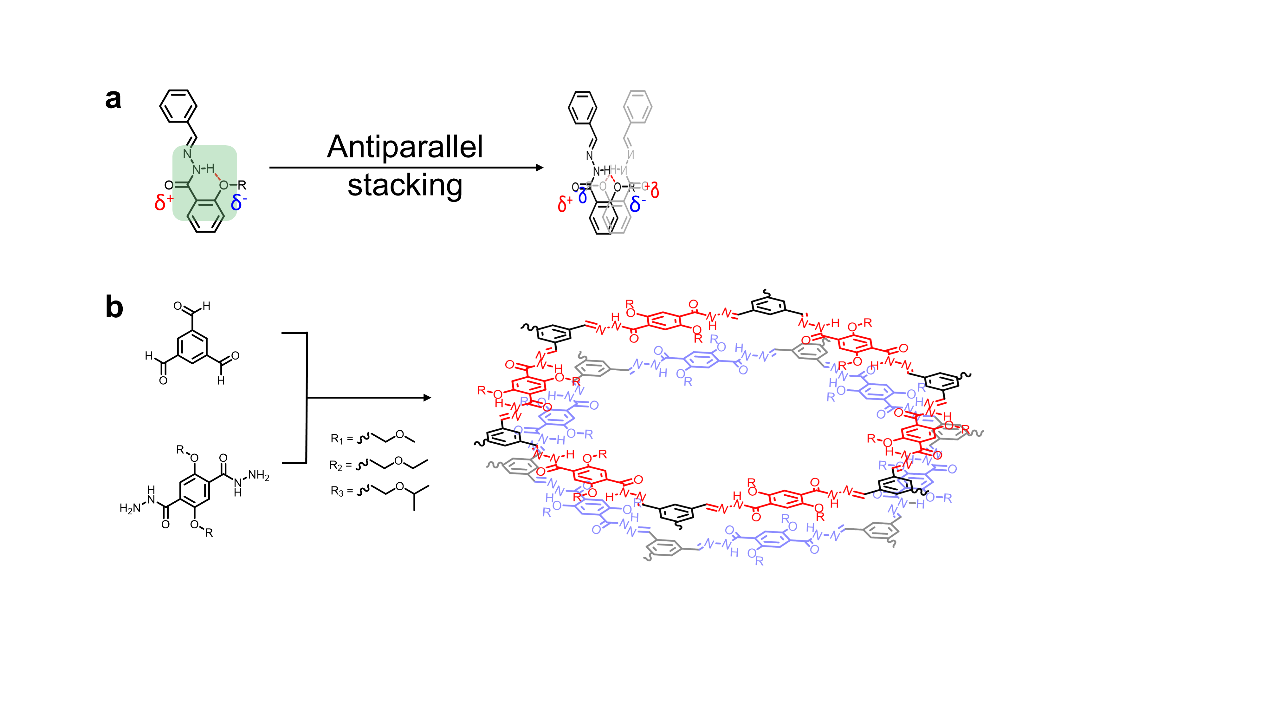


**Figure S1.** Dipole-induced antiparallel stacking (a) and synthesis of antiparallel stacking COF-PEO-Me/Et/iPr (b).

**Synthesis** **of** **COF-PEO-Me/Et/iPr Gel**

**Glue A configuration：**TF (16.0 mg, 0.10 mmol) were weighed into a glass ampoule (volume of ca. 10 mL, body length of 9 cm, neck length of 4.5 cm), then a catalytic amount of acetic acid and 1 mL of DMF were added to the bottle and Glue A was obtained after complete dissolution. (DMF can be replaced with the following solvents: DMAc, THF, CAN and Diox).

**Glue B configuration：**PEO-Me (51.3 mg, 0.15 mmol) were weighed into a glass ampoule (volume of ca. 10 mL, body length of 9 cm, neck length of 4.5 cm), then 1 mL of H_2_O were added to the bottle and Glue B was obtained after complete dissolution.

**Gel preparation：**Quickly mix Glue A& Glue B, pour into mold and COF-PEO-Me was obtained. COF-PEO- Et/iPr Gel was prepared in agreement with COF-PEO-Me.

**Synthesis** **of** **COF-PEO-Me/Et/iPr Sol**

**Solution A configuration：**PEO-Me (51.3 mg, 0.15 mmol) were weighed into a glass ampoule (volume of ca. 10 mL, body length of 9 cm, neck length of 4.5 cm), then 1 mL of CH_2_Cl_2_ were added to the bottle and Glue B was obtained after complete dissolution. (CH_2_Cl_2_ can be replaced with the following solvents: o-DCB and Mesitylene).

**Sol preparation：**Solution A was added to Glue A and reacted for 5-10 min at room temperature to obtain COF-PEO-Me sol. COF-PEO- Et/iPr Sol was prepared in agreement with COF-PEO-Me.

4. Characterization of monomer and COF materials


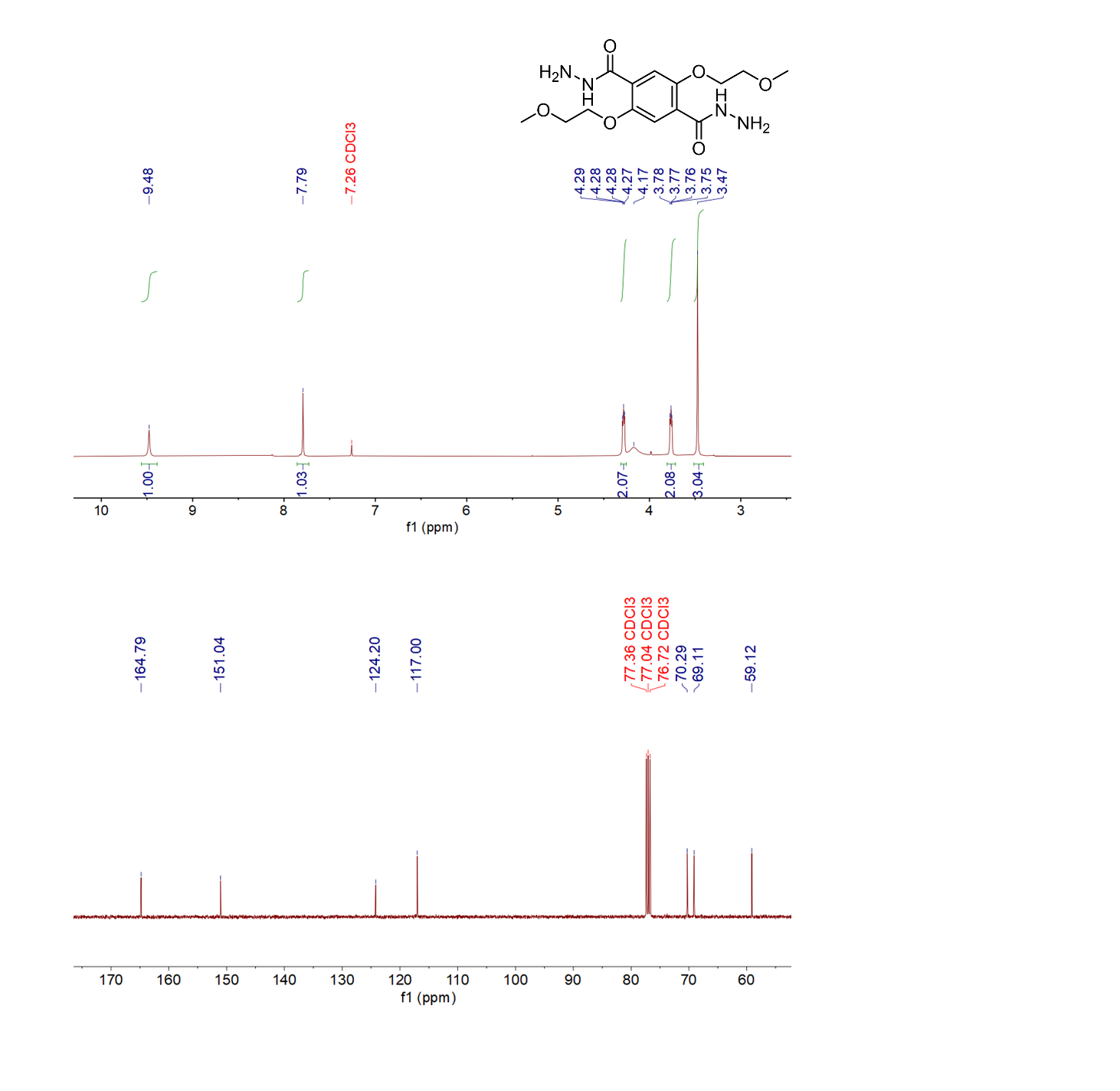


**Figure S2.** ^1^H NMR (CDCl_3_) and ^13^C NMR (CDCl_3_) spectra of **PEO-Me**.


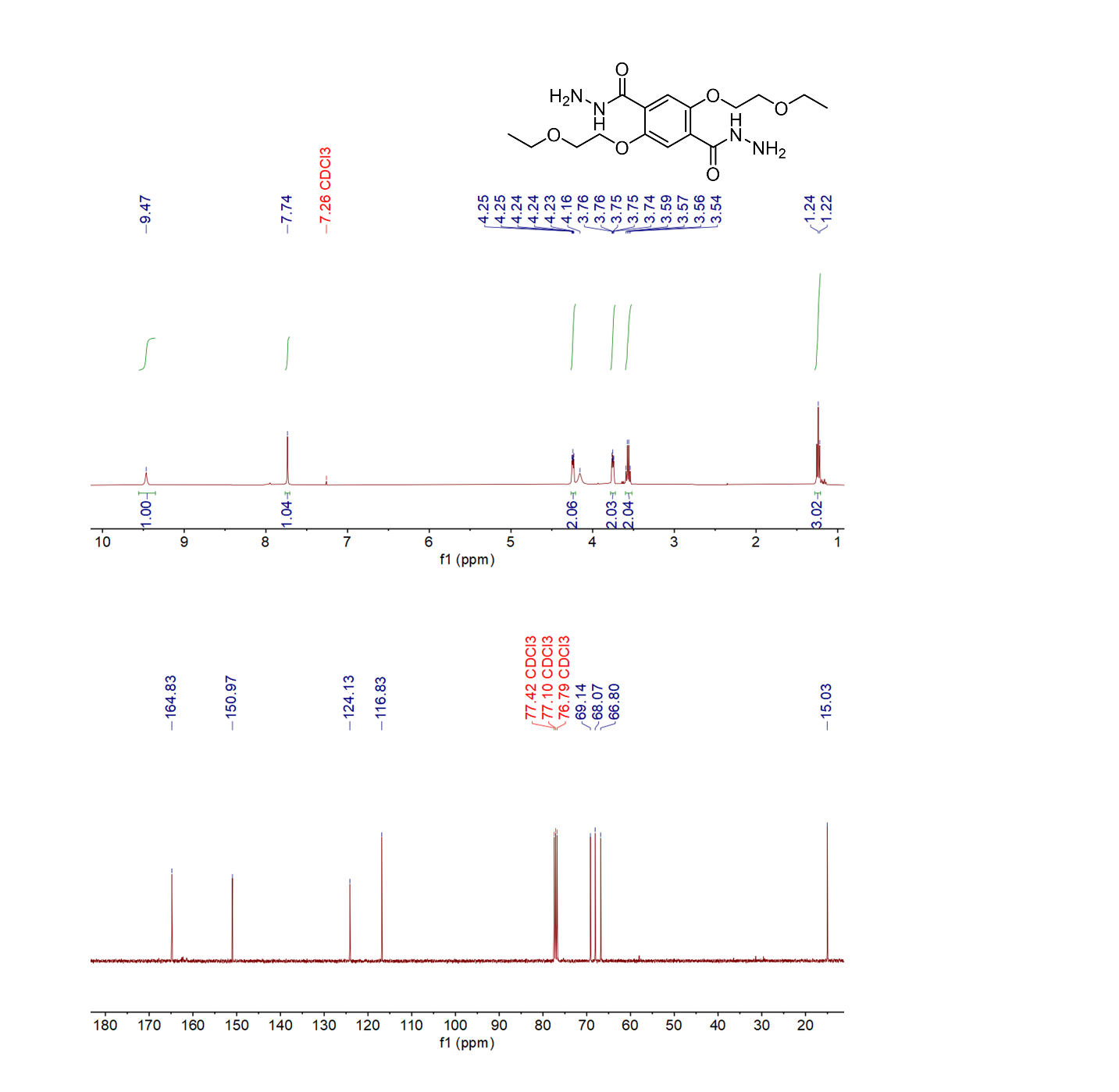


**Figure S3.** ^1^H NMR (CDCl_3_) and ^13^C NMR (CDCl_3_) spectra of **PEO-Et**.


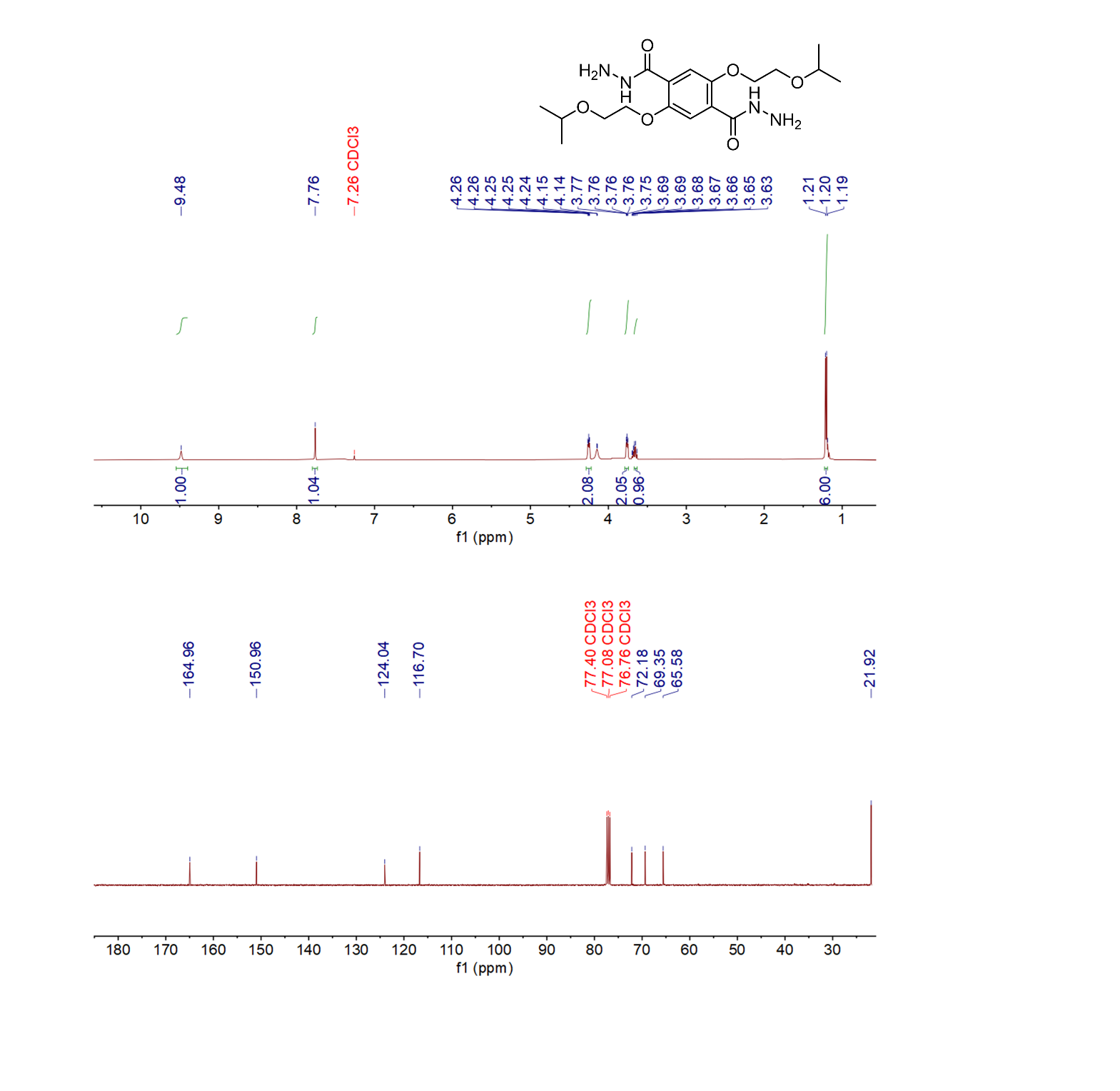


**Figure S4.** ^1^H NMR (CDCl_3_) and ^13^C NMR (CDCl_3_) spectra of **PEO-iPr**.


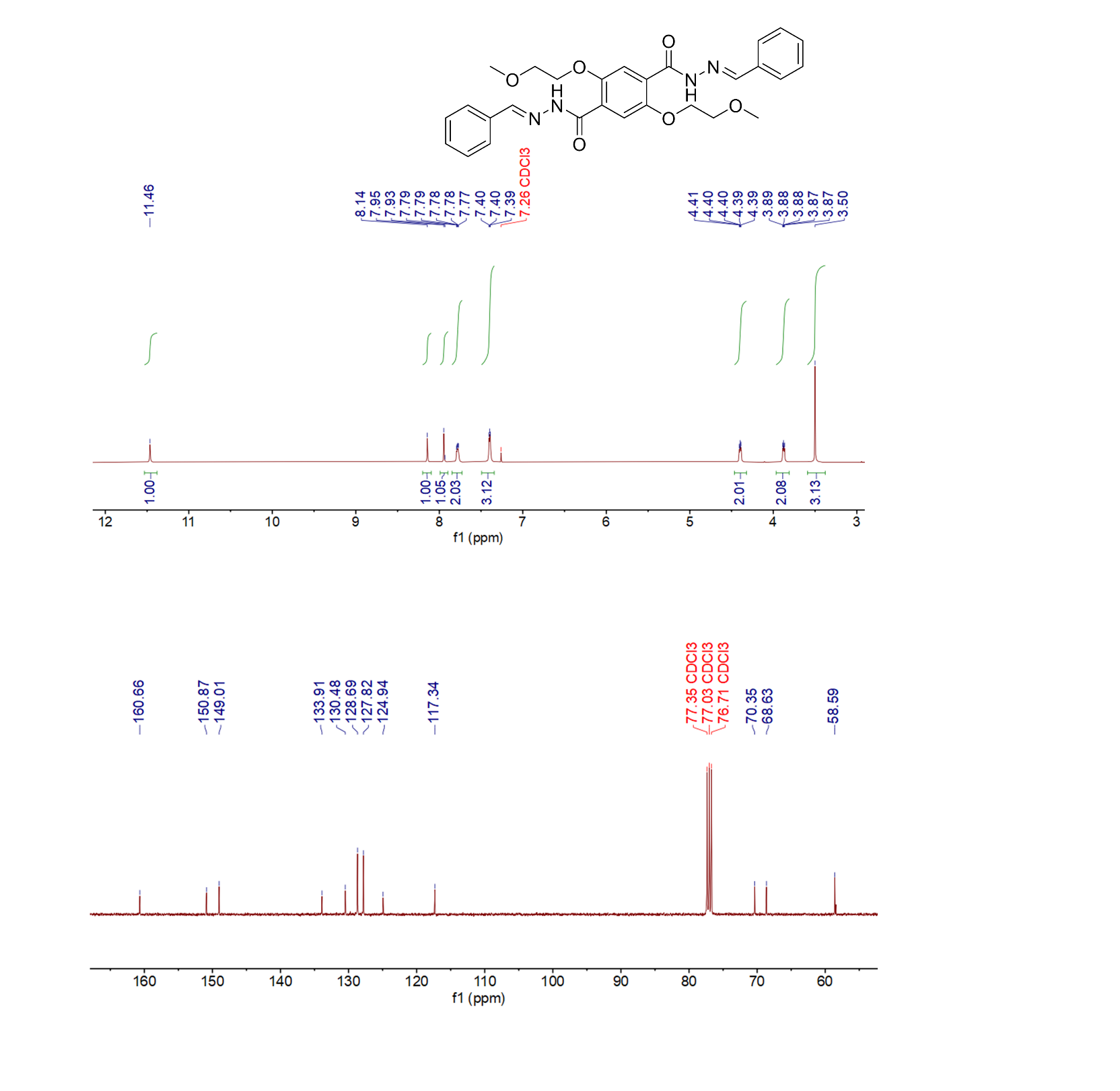


**Figure S5.** ^1^H NMR (CDCl_3_) and ^13^C NMR (CDCl_3_) spectra of **Model 1**.


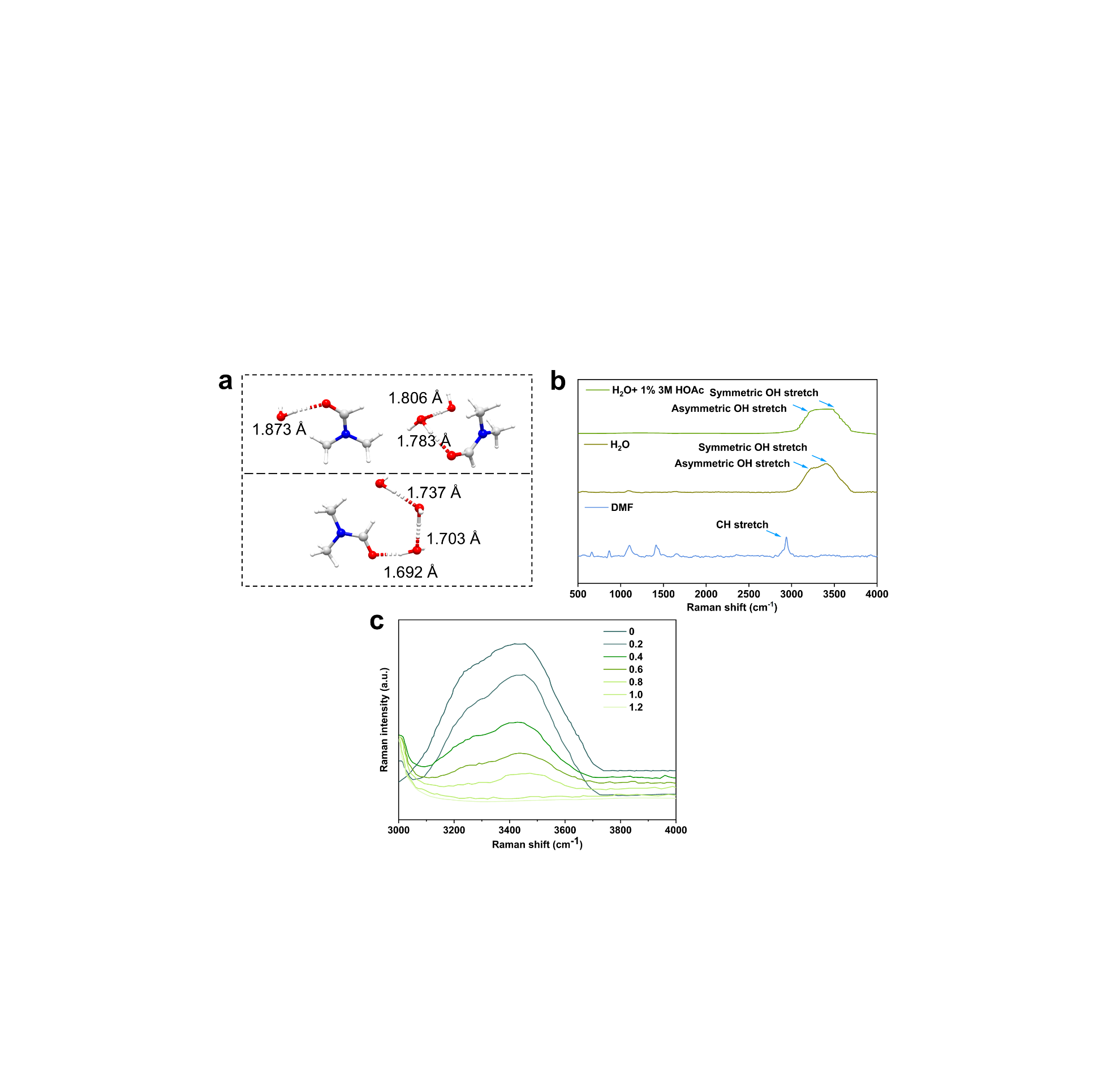


**Figure S6.** (a) Hydrogen bonding between different components of water and DMF; (b) Raman spectra of water and DMF; (c) Raman spectra of different water and DMF fractions.


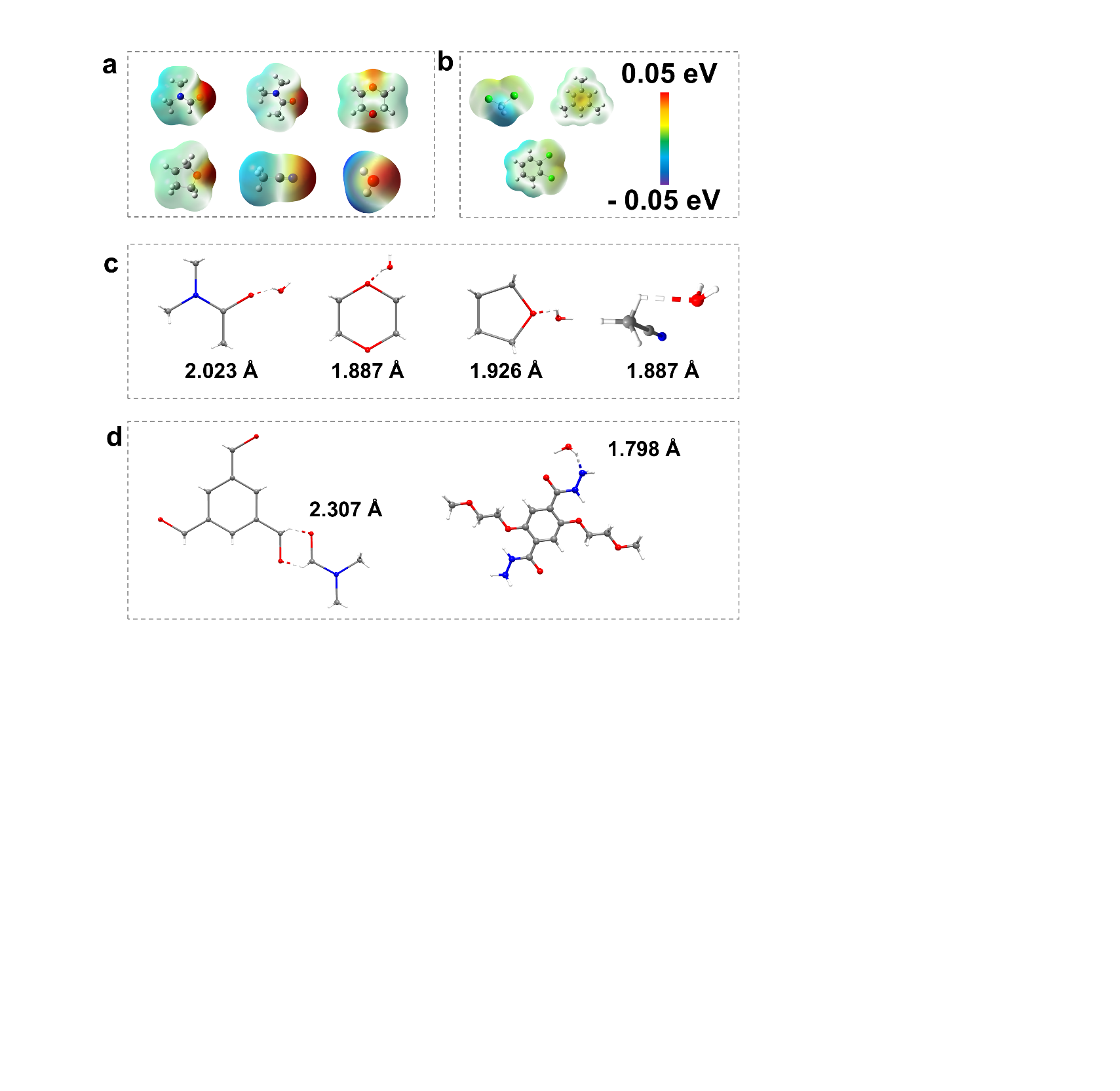


**Figure S7.** Electrostatic diagrams of (a) Glue A and (b) Solution A.(c) Hydrogen bonding between water molecules and solvents such as DMAc. (d) Hydrogen bonding between monomer and solvent.


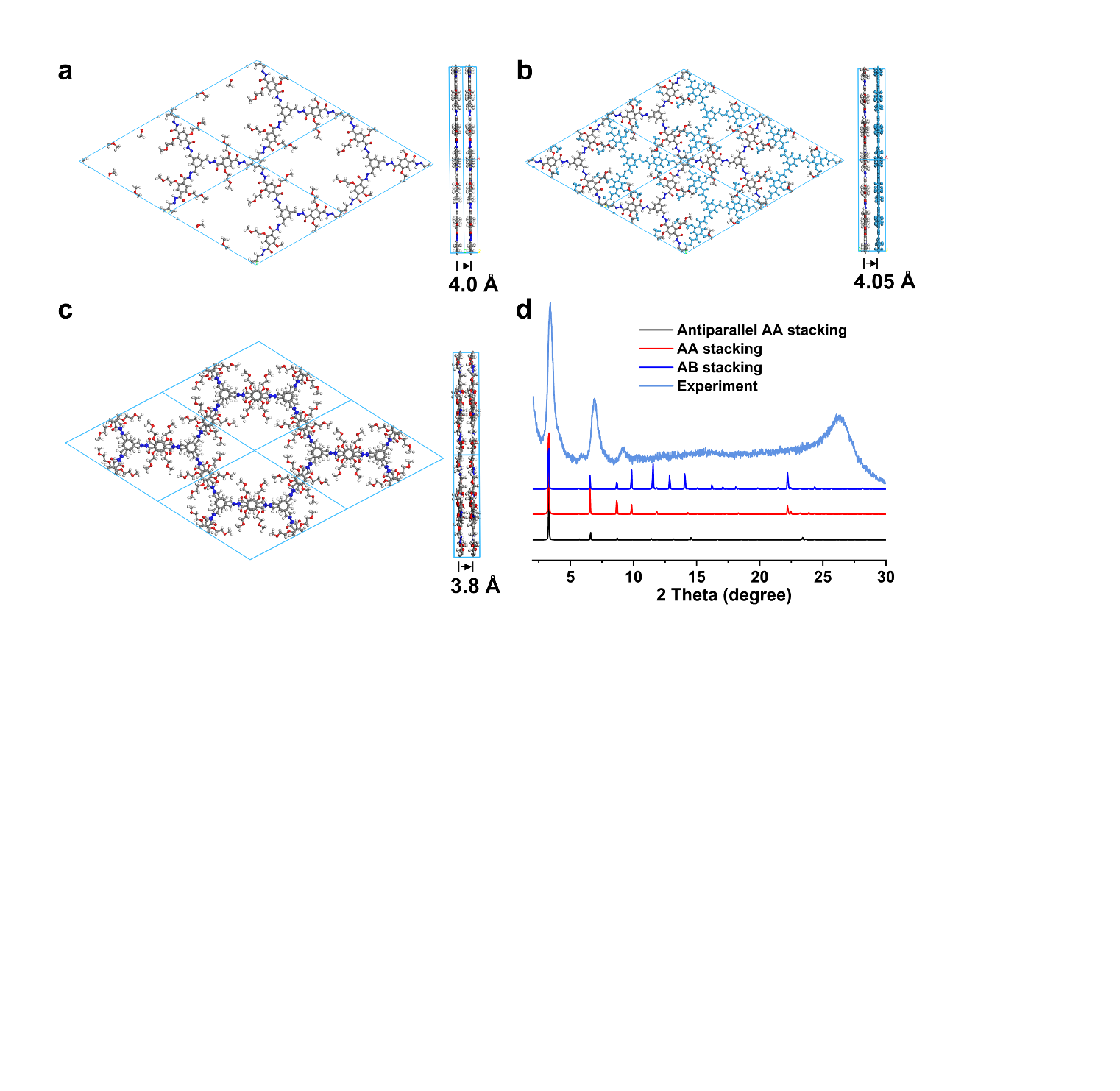


**Figure S8.** The calculated structure of COF-PEO-Me: (a) AA stacking; (b) AB stacking; (c) Antiparallel AA stacking. (d) PXRD patterns of COF-PEO-Me.


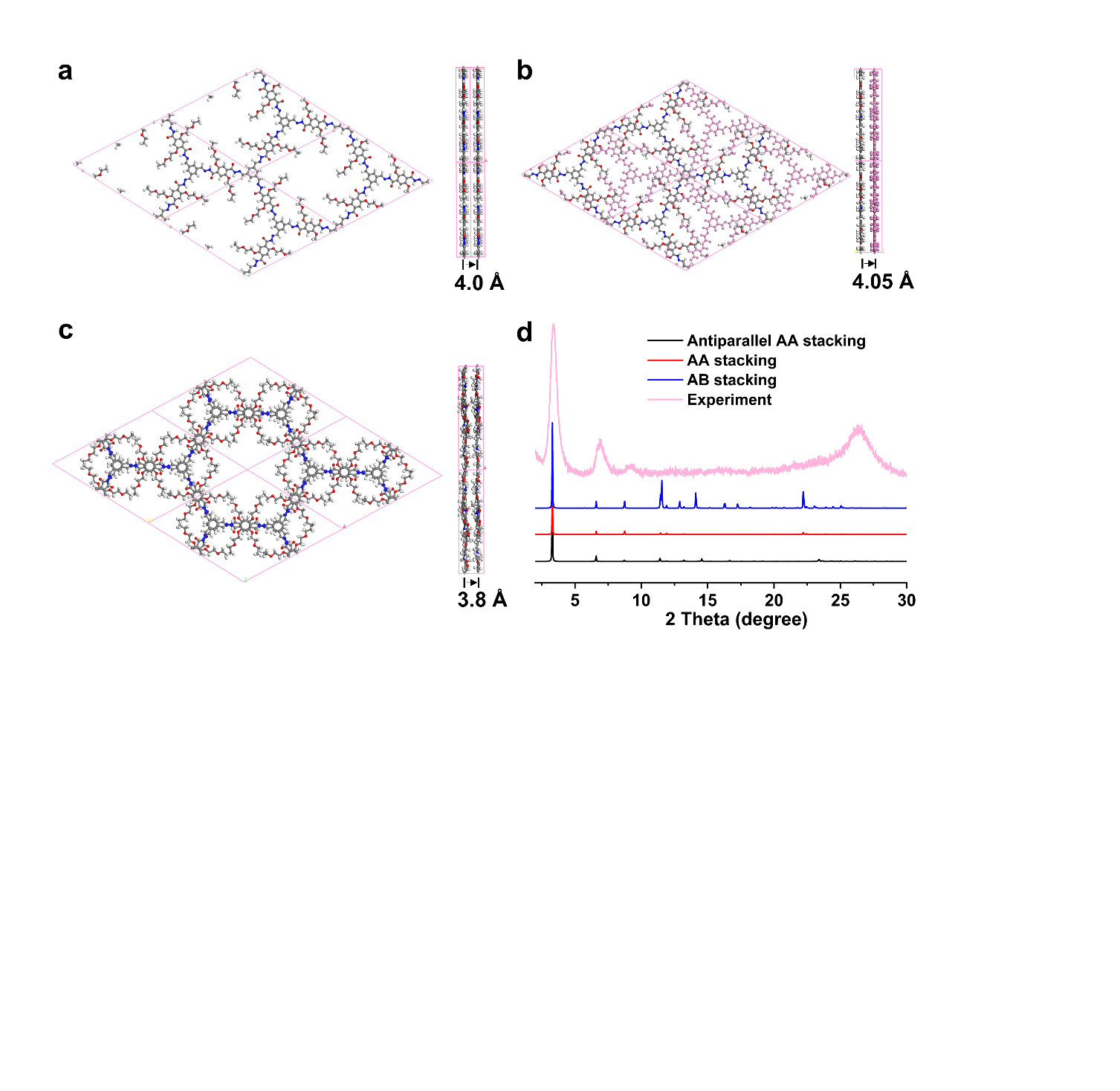


**Figure S9.** The calculated structure of COF-PEO-Et: (a) AA stacking; (b) AB stacking; (c) Antiparallel AA stacking. (d) PXRD patterns of COF-PEO-Et.


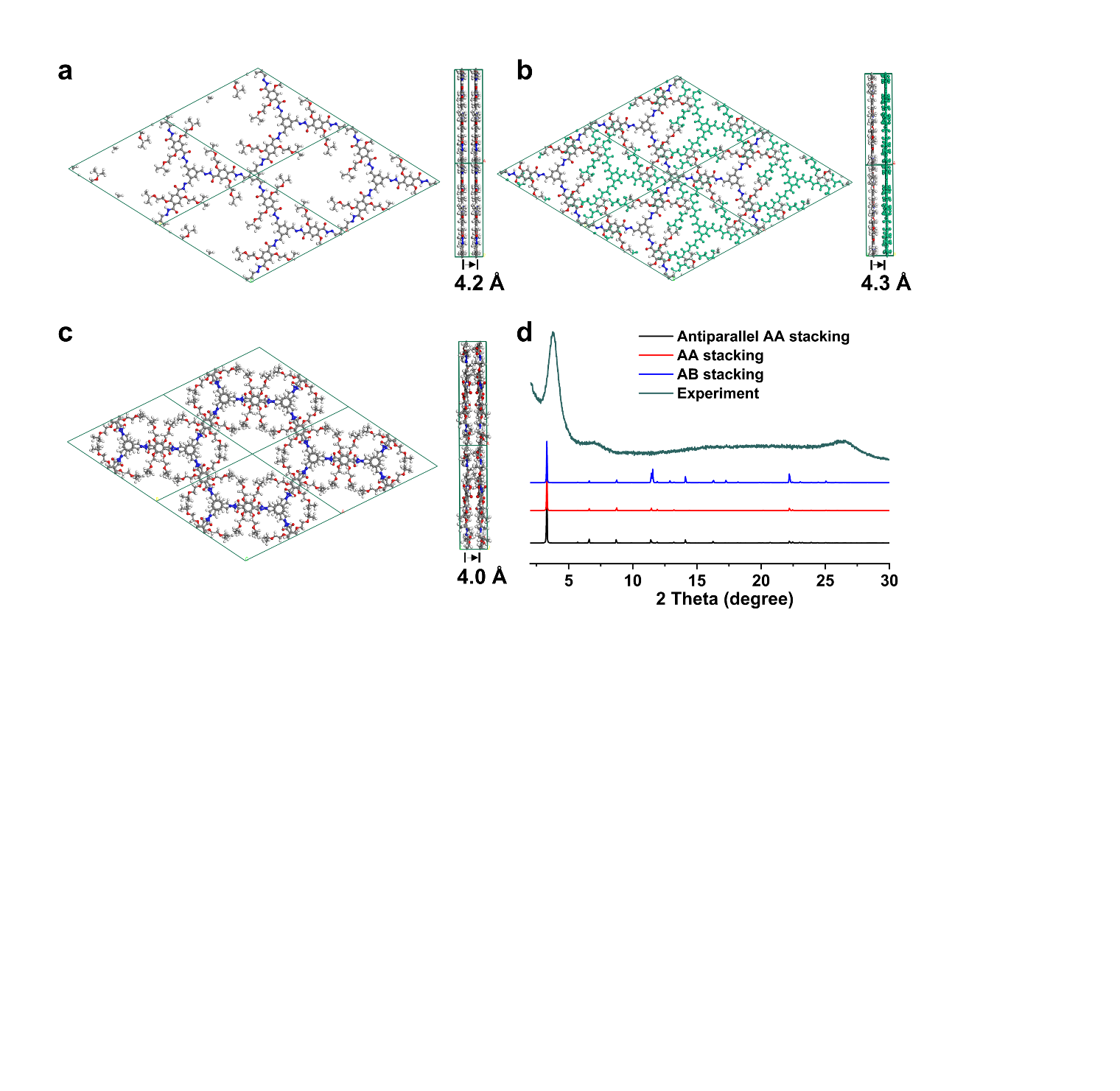


**Figure S10.** The calculated structure of COF-PEO-iPr: (a) AA stacking; (b) AB stacking; (c) Antiparallel AA stacking. (d) PXRD patterns of COF-PEO-iPr.


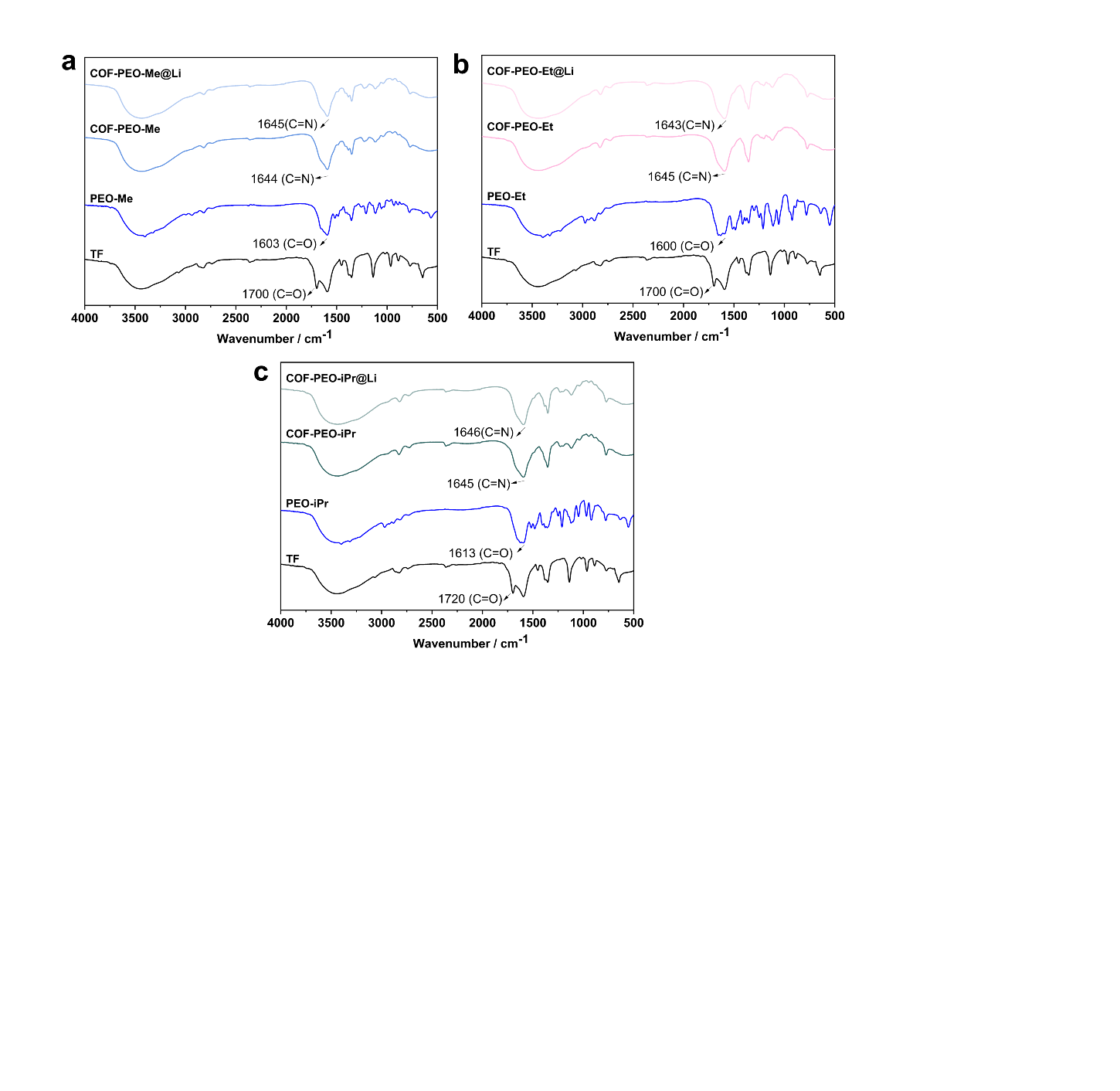


**Figure S11.** FT-IR spectra of COF-PEO-Me (a), COF-PEO-Et (b) and COF-PEO-iPr (c).


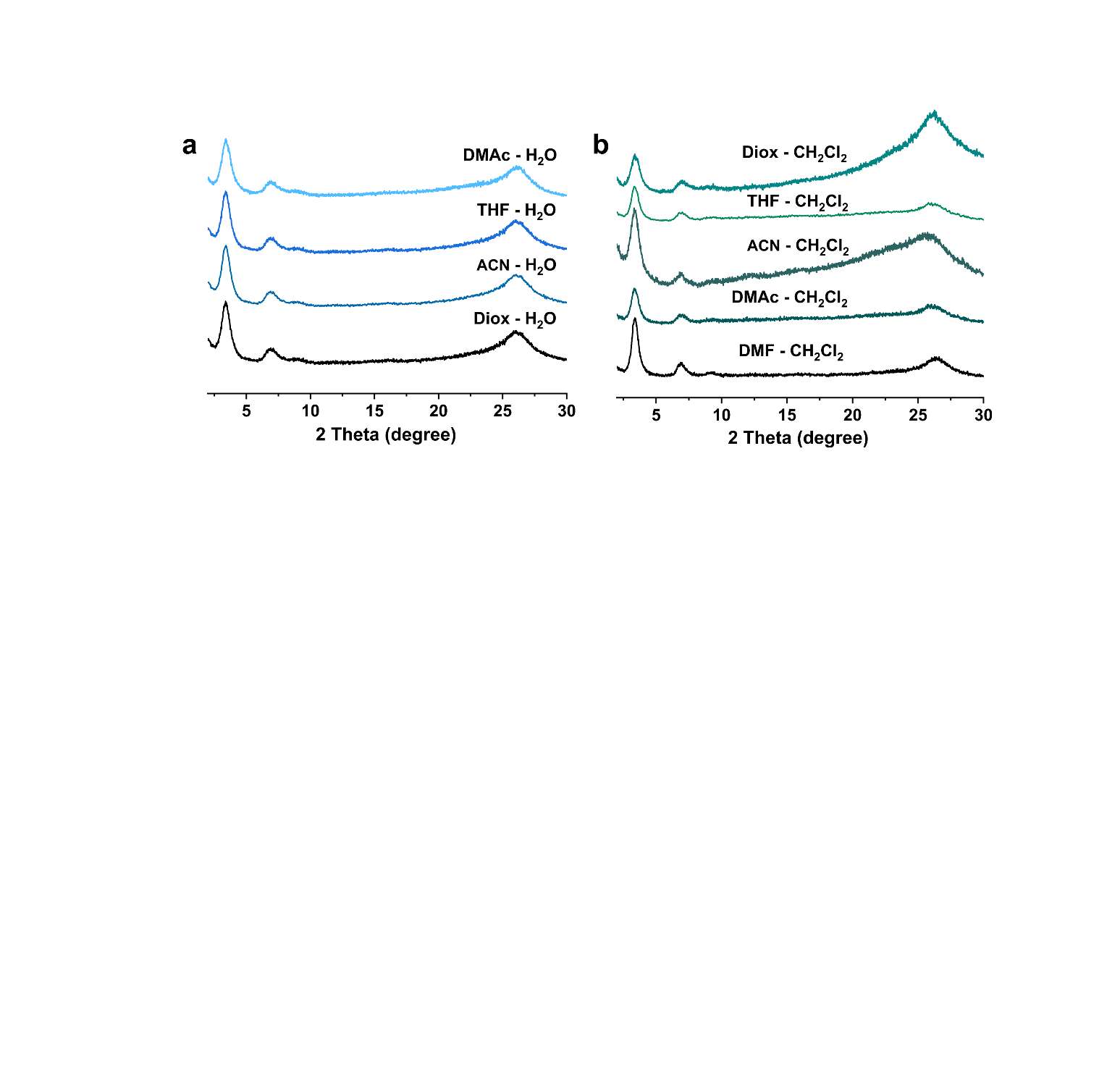


**Figure S12.** PXRD patterns of COF-PEO-Me: (a) Gel; (b) Sol.


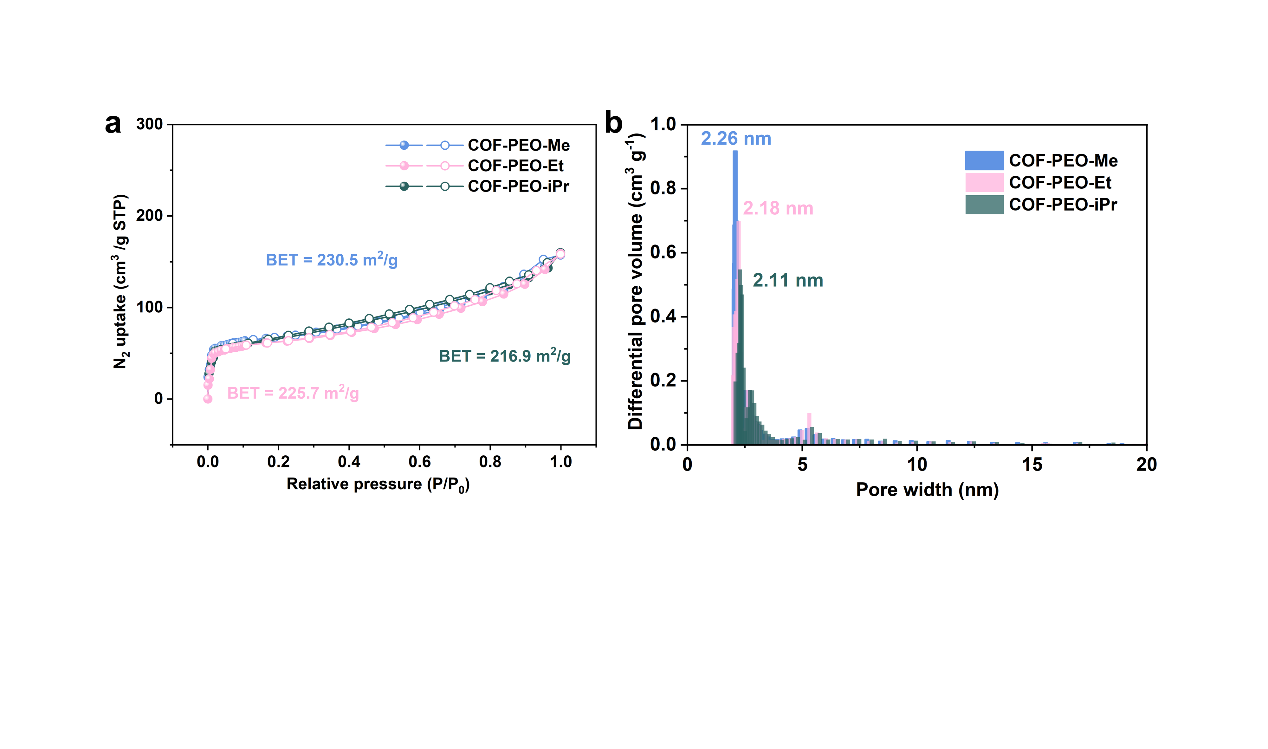
**Figure S13.** N_2_ adsorption isotherm of COF-PEO-Me/Et/iPr.


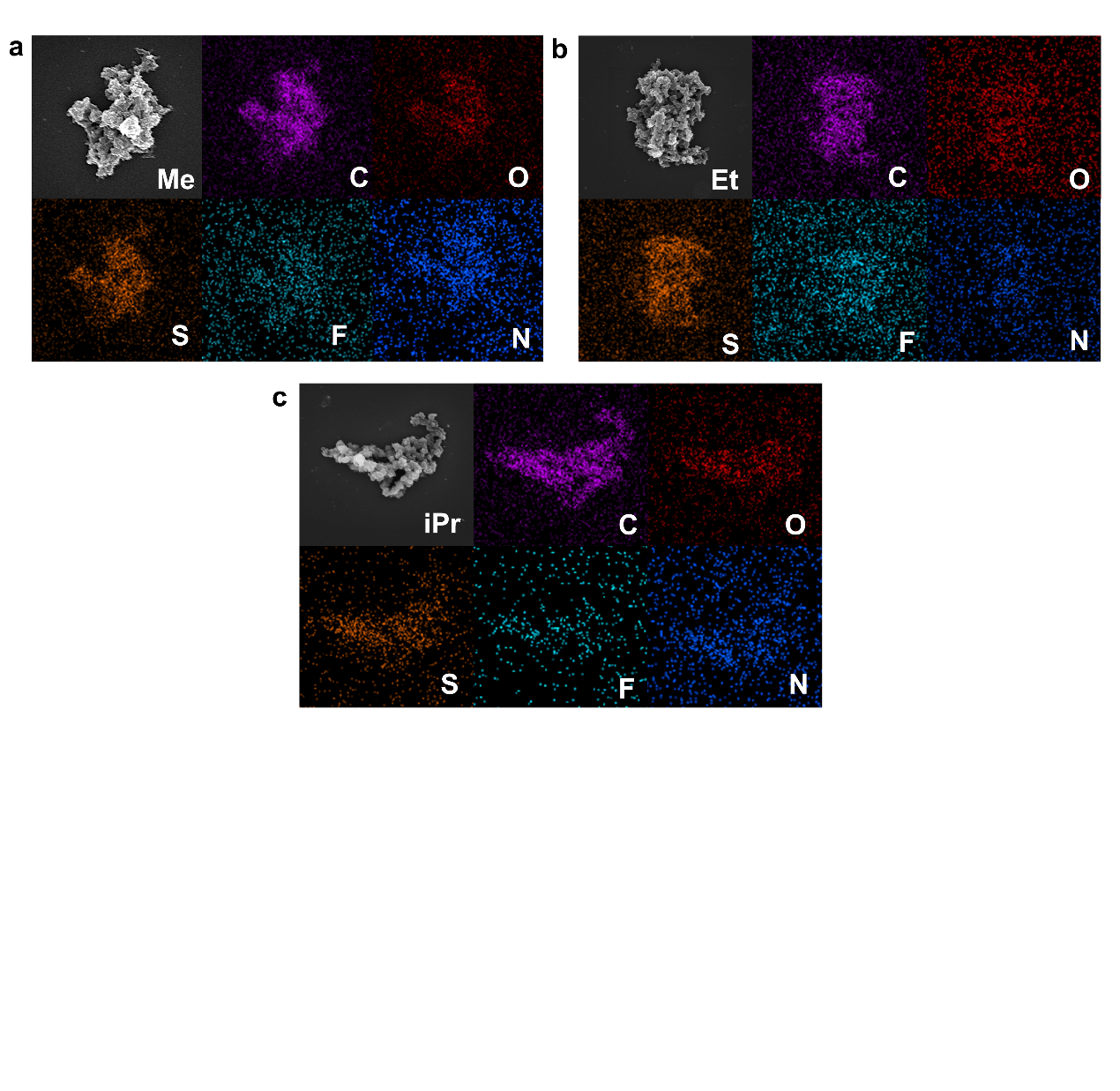


**Figure S14.** EDS images of COF-PEO-Me (a), COF-PEO-Et (b) and COF-PEO-iPr (c) after electrolyte replacement.


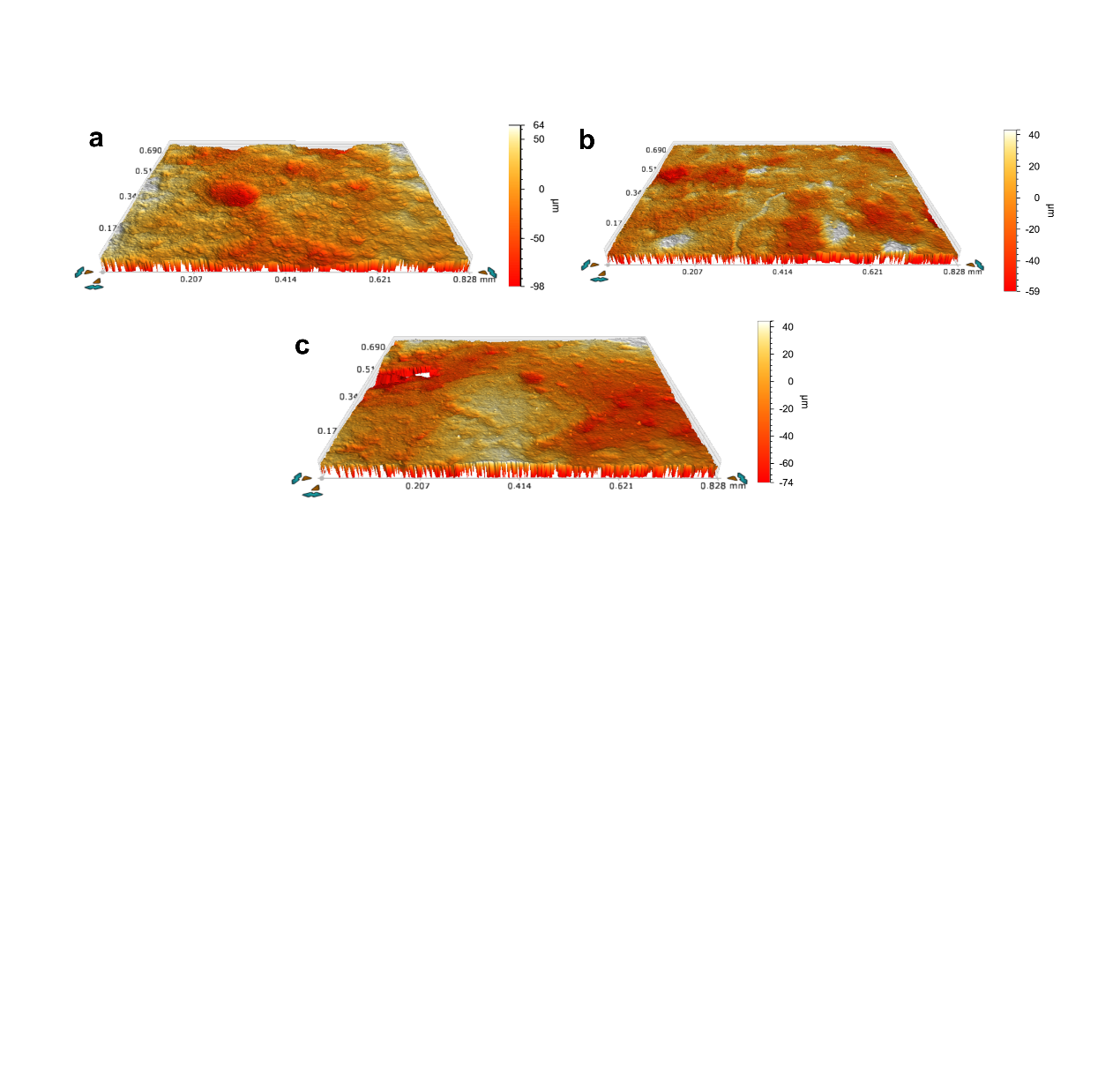


**Figure S15.** Scanning laser confocal microscope images of COF-PEO-Me (a), COF-PEO-Et (b) and COF-PEO-iPr (c).


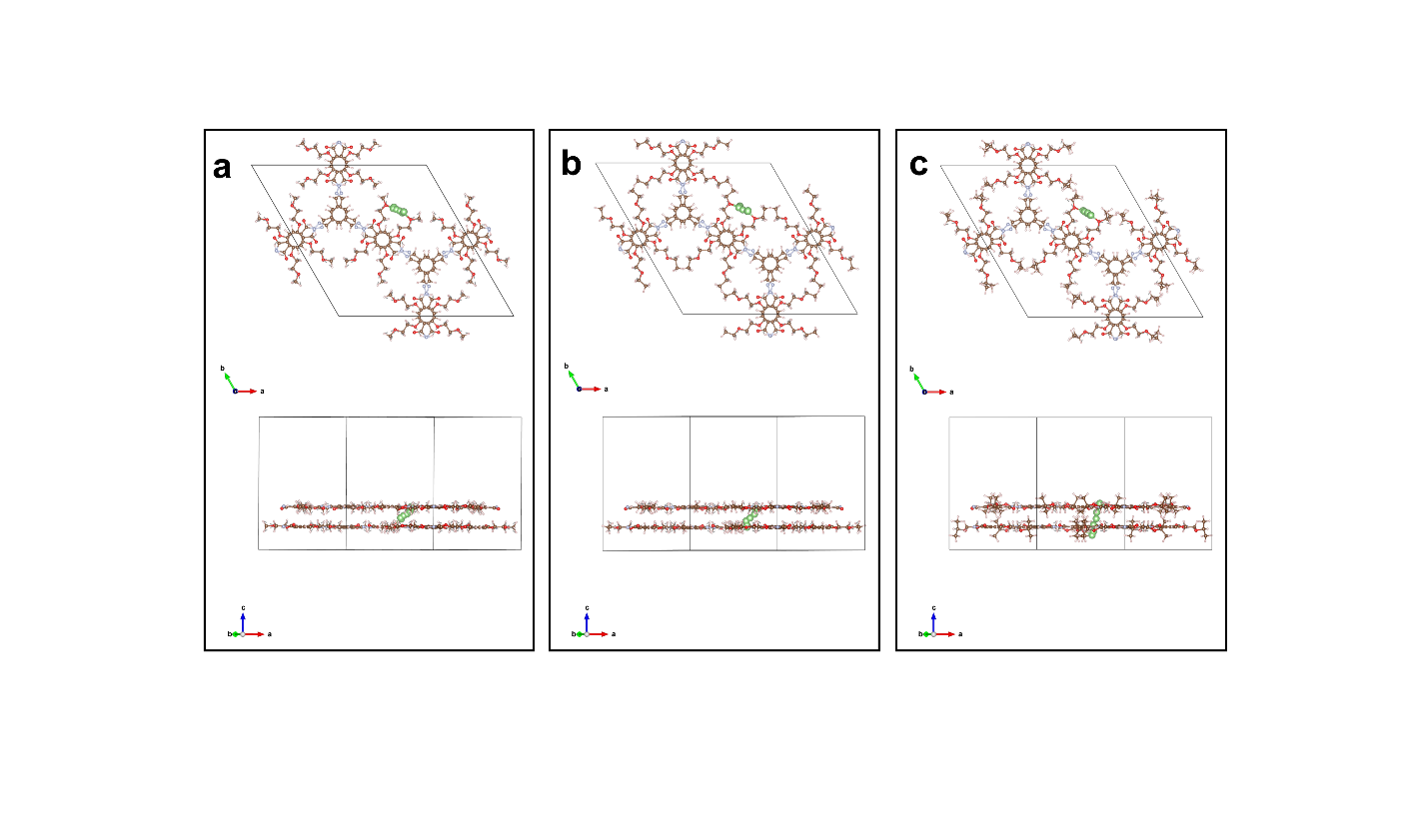
**Figure S16.** The corresponding structural model of COF-PEO-Me (a), COF-PEO-Et (b) and COF-PEO-iPr (c).


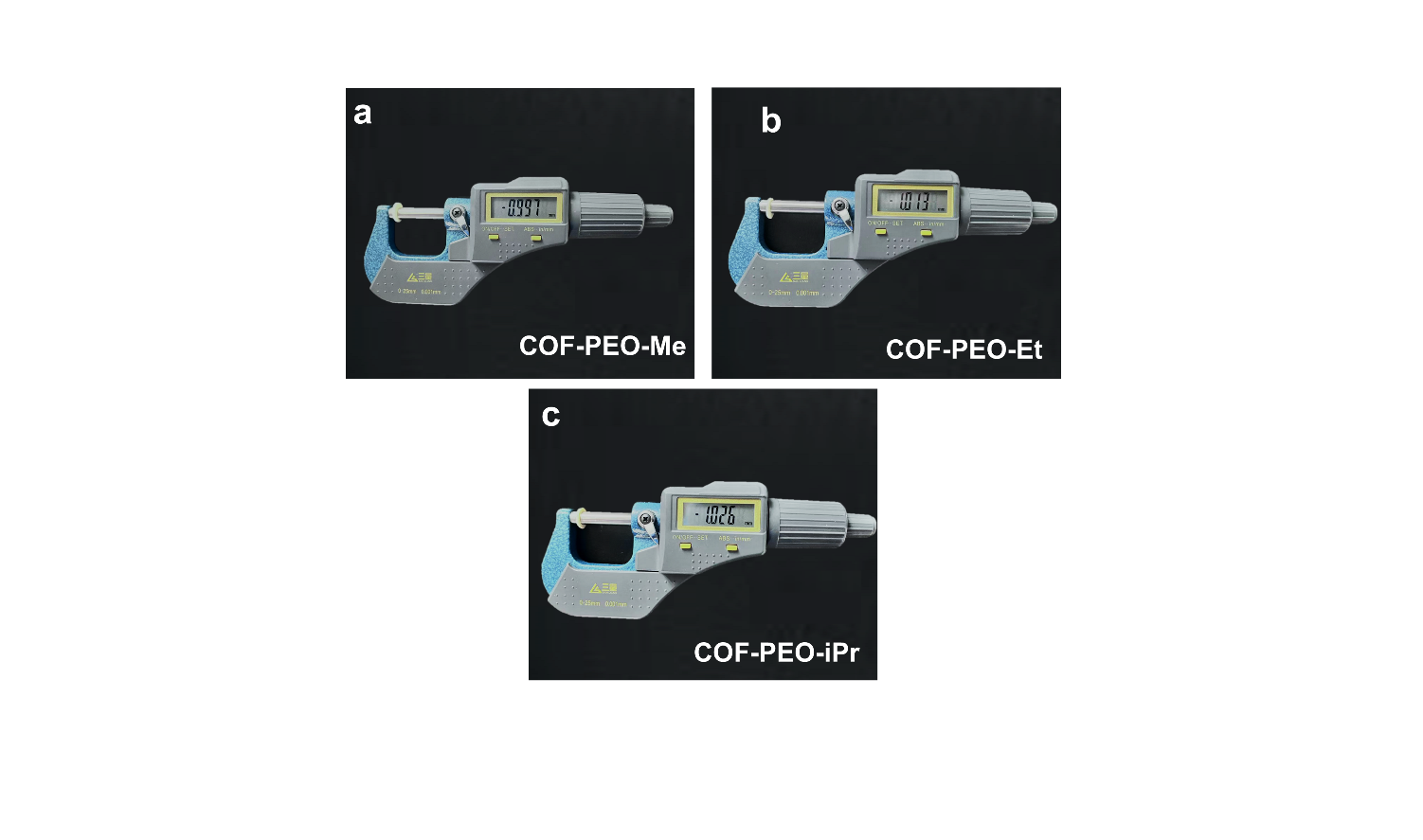
**Figure S17.** The thickness of COF-PEO-Me (a), COF-PEO-Et (b) and COF-PEO-iPr (c).


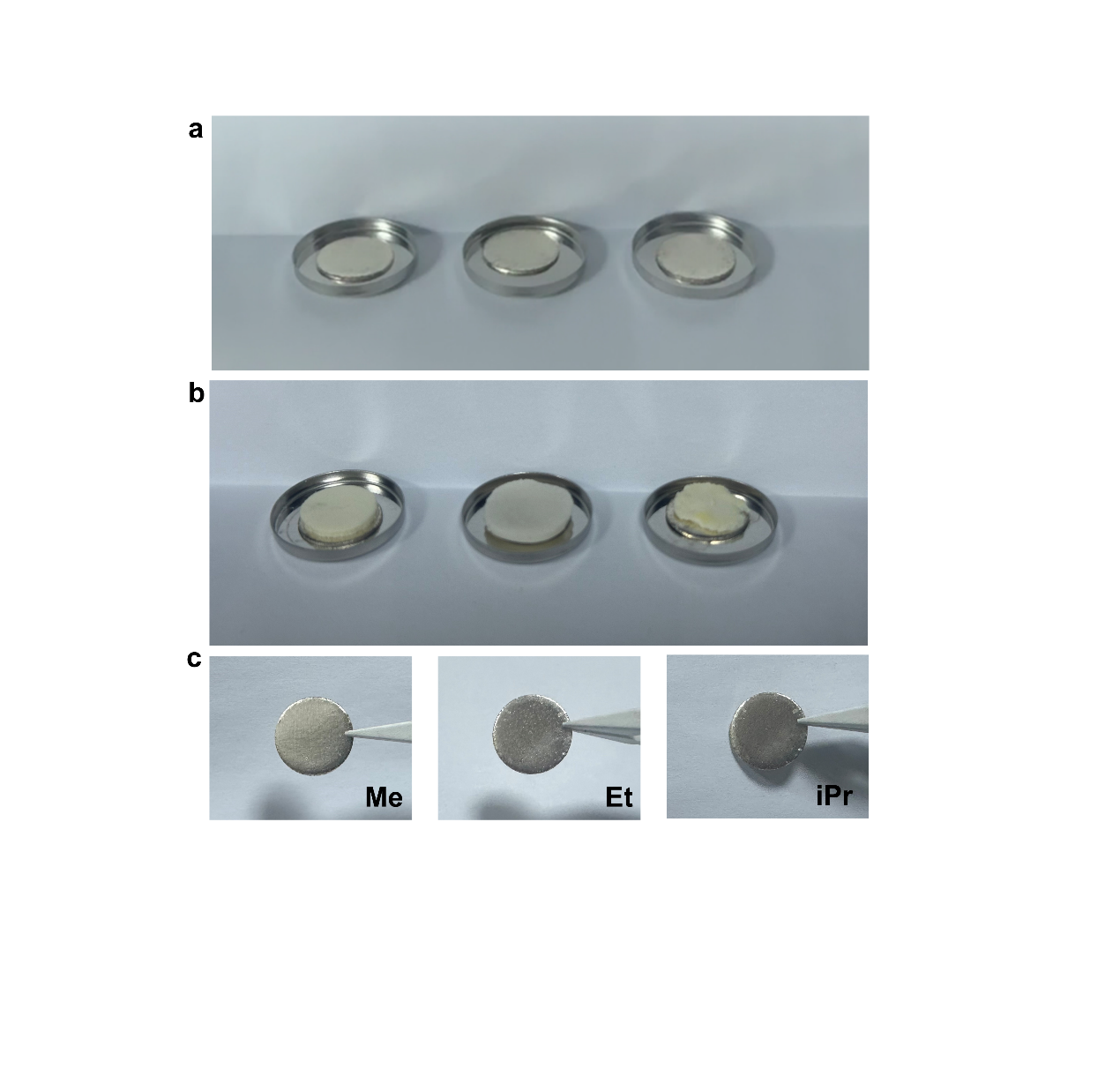


**Figure S18.** Digital photo of (a) pristine lithium metal electrode. (b) Precursor covered with lithium metal and left to stand for 24 hours. Digital photo of (c) lithium metal after gel removal.


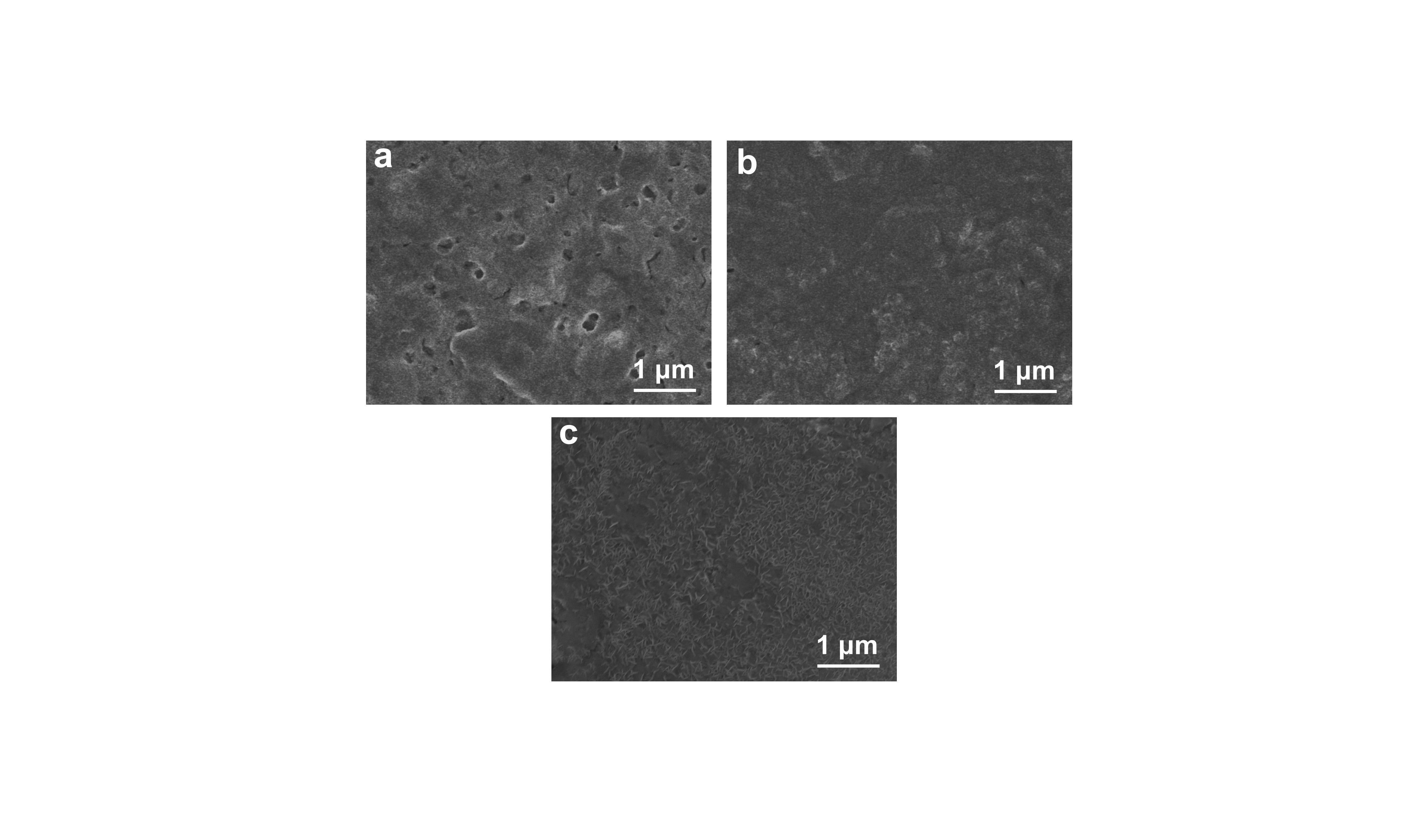
**Figure S19.** SEM images of lithium metal anode after cycling from cells with different electrolytes: COF-PEO-Me (a), COF-PEO-Et (b) and COF-PEO-iPr (c).


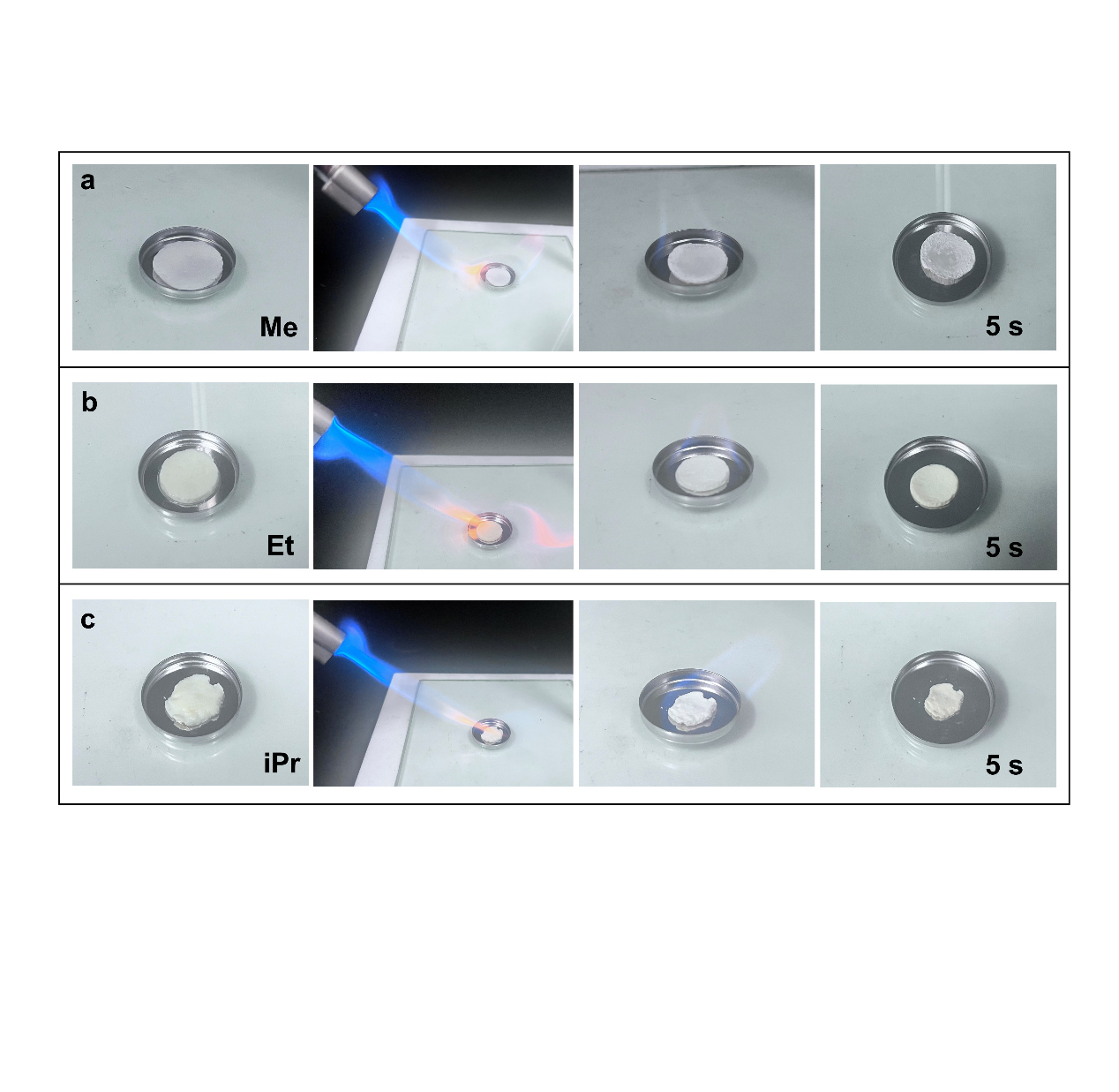


**Figure S20.** Combustion reaction of COF-PEO-Me (a), COF-PEO-Et (b) and COF-PEO-iPr (c).

**Table S1.** Digital images of Sol prepared with different solvents.


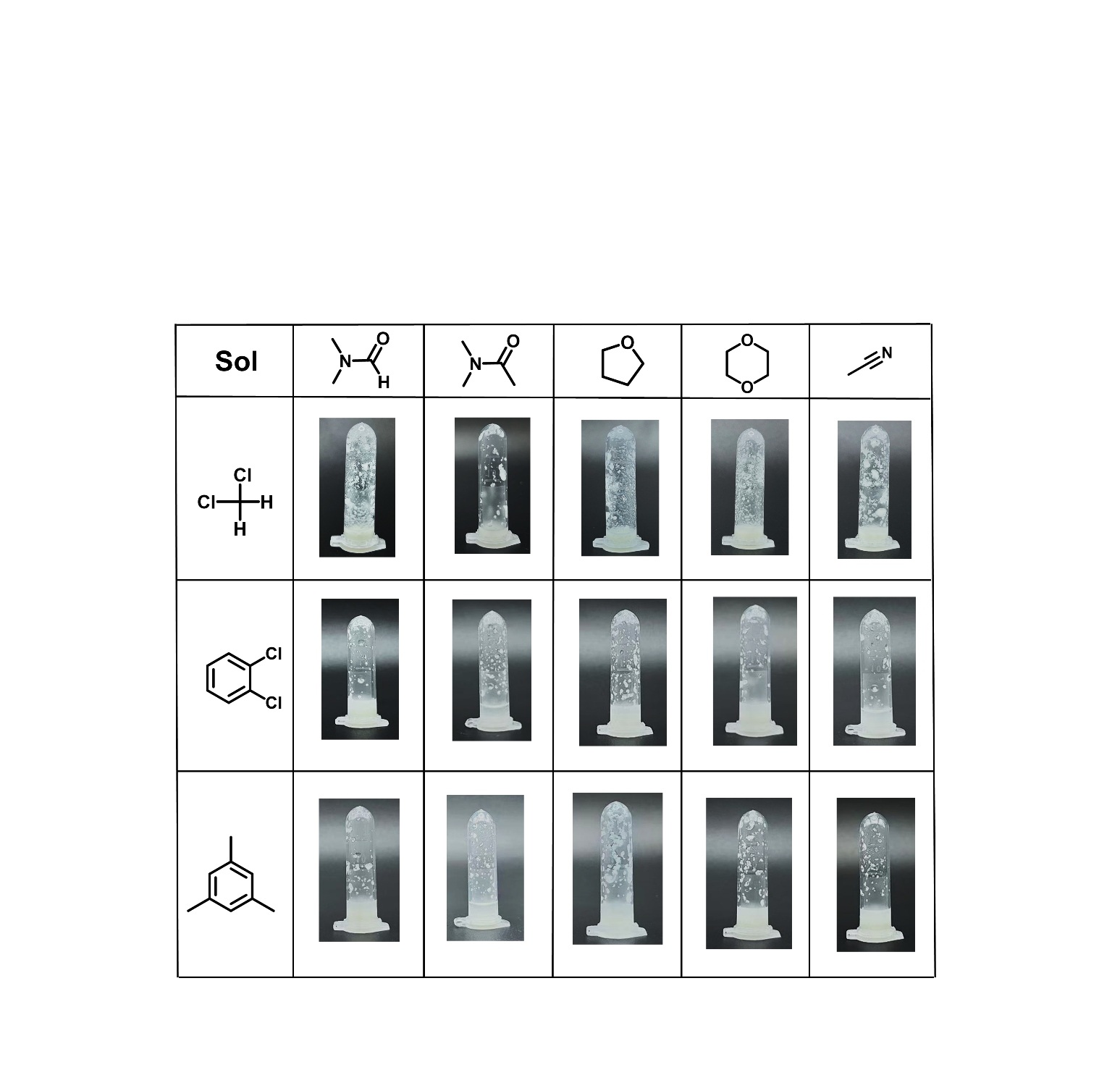


**Reference**

[1] Perdew, J. P.; Burke, K.; Ernzerhof, M. Generalized Gradient Approximation Made Simple. *Phys. Rev. Lett.* **1996**, *77*, 3865.

[2] Grimme, S.; Antony, J.; Ehrlich, S.; Krieg, H. A Consistent and Accurate Ab Initio Parametrization of Density Functional Dispersion Correction (DFT-D) for the 94 Elements H-Pu. *J. Chem. Phys.* **2010**, *132*, 154104.

[3] Monkhorst, H. J.; Pack, J. D. Special Points for Brillouin-Zone Integrations. *Phys. Rev. B* **1976**, *13*, 5188.

[4] Kresse, G.; Furthmüller, J. Efficient Iterative Schemes for Ab Initio Total-Energy Calculations Using a Plane-Wave Basis Set. Phys. Rev. B Condens. *Matter Mater. Phys.* **1996**, *54*, 11169.

[5] Kresse, G.; Furthmüller, J. Efficiency of Ab-Initio Total Energy Calculations for Metals and Semiconductors Using a Plane-Wave Basis Set. *Comput. Mater. Sci.* **1996**, *6*, 15.

[6] Meng, F.; Fu, X.; Ni, Y.; Sun, J.; Li, Z. Biomimetic polypeptides with reversible pH-dependent thermal responsive property. *Polymer*, **2017**, 1*18*, 173-179.
